# Synthesis and Properties of 1*H*-Pyrrolo[3′,2′:3,4]fluoreno[9,1-*gh*]quinolines and 7*H*-Pyrrolo[2′,3′,4′:4,10]anthra[1,9-*fg*]quinolines

**DOI:** 10.3390/molecules30122615

**Published:** 2025-06-16

**Authors:** Aleksandra Khomutetckaia, Peter Ehlers, Alexander Villinger, Peter Langer

**Affiliations:** Institut für Chemie, Universität Rostock, A.-Einstein-Str. 3a, 18059 Rostock, Germany

**Keywords:** PAHs, catalysis, cyclization, CH activation, heterocycles, palladium, regioselectivity

## Abstract

We report the synthesis of pyrrolo[3′,2′:3,4]fluoreno[9,1-*gh*]quinoline and pyrrolo[2′,3′,4′:4,10]anthra[1,9-*fg*]quinoline derivatives. This novel class of N-doped polycyclic heteroaromatic compounds was synthesized by a site-selective cross-coupling reaction followed by acid-mediated cycloisomerization and Pd-catalyzed CH arylation as the final ring-closing reactions. Preliminary optical and aromatic properties were studied by means of steady-state absorption and fluorescence spectroscopy and DFT calculation. Special emphasis was placed on the impact of ring alternation and position of the N-doping within the scaffold.

## 1. Introduction

In the past, polycyclic aromatic compounds (PAHs) were merely known for their cancerogenic properties, which remain a subject of debate [1,2,3]. However, this class of compounds has nonetheless attracted widespread attention in the field of materials science [1,2,4,5,6,7,8]. These compounds offer unique (opto-)electronic properties accompanied by improved processability and tunability compared to their inorganic counterparts. Hence, PAH-based devices have been successfully applied as organic field-effect transistors (OFETs) [5,9,10,11], organic light-emitting diodes (OLEDs) [12,13,14], and organic photovoltaic cells (OPVs) [15,16,17].

The introduction of heteroatoms into the π-conjugated scaffold is an efficient strategy for altering the inherent properties of compounds and allows for the fine-tuning of their physical properties [18,19,20]. For instance, the incorporation of heteroatoms affects HOMO and LUMO energies and, consequently, optical and electrochemical properties and redox behavior. It influences coordination ability and acid–base chemistry, as well as bioactivity, to name but a few. In particular, nitrogen-containing PAHs have gained considerable attention due to their ability to change electronic properties depending on the nature of N-doping. The incorporation of imine-like N-atoms within the scaffold stabilizes the HOMO and LUMO energies, while amine-like nitrogen atoms improve the electron-donating properties of the polycyclic compound. Alternatively, alterations in ring size can influence the (opto-)electronic properties of PAHs and, hence, are a subject of intense research. For instance, the incorporation of five-membered rings can lead to unusual (photo-)physical properties and high electron affinities, similar to those of fullerene and its subunits. In addition, the incorporation of five-membered rings can lead to curved or bowl-shaped structures with intriguing properties, stemming from distorted orbital orientations [21,22,23,24].

As part of our ongoing interest in the synthesis of N-doped PAHs with alternating ring systems [25,26,27,28], we herein report the synthesis of pyrroloindeno- and pyrroloanthraquinoline derivatives. To the best of our knowledge, such structural motifs have not been described in the literature, and related carbocyclic PAHs are only rarely synthesized or detected as pollution components from domestic burning of coal and wood [29,30,31,32,33,34,35,36,37]. The synthetic strategy is based on the site-selective cross-coupling reaction of respective dibromopyridines followed by acid-mediated cycloisomerization. Pd-catalyzed CH arylation gives the final product in high yield [38].

## 2. Results and Discussion

**Scheme 1:**
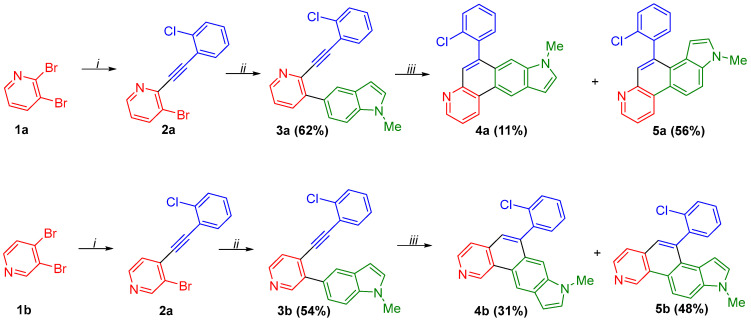
Synthesis strategy for 1-methyl-1*H*-pyrrolo[3′,2′:3,4]fluoreno[9,1-*gh*]isoquinoline and 1-methyl-1*H*-pyrrolo[2′,3′,4′:4,10]anthra[9,1-*gh*]isoquinoline derivatives. Conditions: *i* (for **2a**), **1a** (1.0 equiv.), 1-Chloro-2-ethynylbenzene (1.2 equiv.), Pd(PPh_3_)_4_ (5 mol%), CuI (2 mol%), HN^i^Pr_2_, 40 °C, 1 h; (for **2b**), **1b** (1.0 equiv.), 1-Chloro-2-ethynylbenzene (1.2 equiv.), Pd(PPh_3_)_4_ (10 mol%), CuI (10 mol%), HN^i^Pr_2_, 40 °C, 3 h; *ii*, **2** (1.0 equiv.), 1-methyl-1*H*-indol-5-yl)boronic acid pinacol ester (1.2 equiv.), Pd(PPh_3_)_4_ (5 mol%), K_3_PO_4_ (2.0 equiv.), 1,4-dioxane/H_2_O (6:1), 100 °C, 3 h; *iii*, **3** (1.0 equiv.), MsOH (30 equiv.), 120 °C, 18 h.

Our synthesis started with the site-selective Sonogashira reactions of 2,3-dibromopyridine **1a** and 3,4-dibromopyridine **1b**. Both reactions proceeded selectively at position 2 or 4 of the pyridine ring, respectively, leaving position 3 of the pyridine ring untouched (Scheme 1). Subsequently, position 3 was substituted by the Suzuki–Miyaura reaction using *N*-methylindole-5-boronic acid pinacol ester, affording the arylated products in moderate yields. As a next step, we studied methanesulfonic acid (MsOH)-mediated cycloisomerization to synthesize indoloquinolines and indoloisoquinolines, respectively. The reaction proceeded smoothly but gave a mixture of isomers, which were separated by column chromatography. Both isomers were identified based on their specific H-H correlations determined by ^1^H-, COSY, and NOESY NMR experiments (for details, see SI). The employed reaction conditions led to the formation of **5a** and **5b** as major products. However, the yield of **4a** was strongly diminished due to issues with its separation from **5a** by column chromatography.

**Scheme 2:**
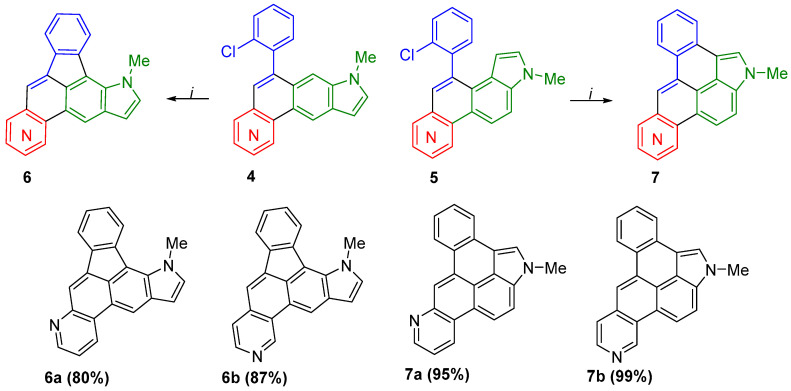
Synthesis of 1-methyl-1*H*-pyrrolo[3′,2′:3,4]fluoreno[9,1-*gh*]isoquinoline (**6**) and 1-methyl-1*H*-pyrrolo[2′,3′,4′:4,10]anthra[9,1-*gh*]isoquinoline derivatives (**7**). Reaction conditions *i:* **4** or **5** (53–120 mg, 1.0 equiv.), PdCl_2_ (0.2 equiv.), PCy_3_ (0.4 equiv.), DBU (3.0 equiv.), NMP, 150 °C, 18 h.

As a final step, we employed both isomers in palladium-catalyzed CH arylation, leading to the corresponding pyrrolo-fluorenoisoquinolines and pyrrolo-anthraisoquinolines. To elucidate suitable reaction conditions, PdCl_2_ was used in a first trial with PCy_3_ as the ligand, DBU as the base, and NMP as the solvent [38]. To our delight, the reaction worked very well, resulting in the desired products in good to excellent yields (Scheme 2). Hence, no further optimization of the reaction conditions was required.

**Figure 1:**
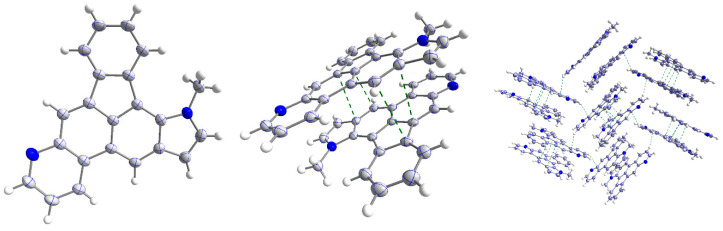
X-ray crystal structure of compound **6a**. (**Left**): single-crystal structure of **6a**; (**Middle**): formed dimers within the lattice with short C⋯C-contacts; (**Right**): herringbone-type alignment of dimers.

Single crystals suitable for X-ray diffraction analysis were grown from a solution of **6a** in dichloromethane and heptane (Figure 1 and Figure S1/Table S1). The molecular structure adopted an almost planar alignment, with deviations from planarity of about 3°and 4° in the two bay regions.

In the crystal lattice, two molecules orientate in opposite directions and form mutual close C-C contacts (3.38 Å and 3.30 Å) between the carbocyclic five-membered rings of the fluorene moiety and the benzene rings neighboring the pyrrole ring. Such dimers align in a herringbone-like pattern, and their respective stacks are stabilized by intermolecular C-H⋯C interactions.

## 3. Physical Properties

**Figure 2:**
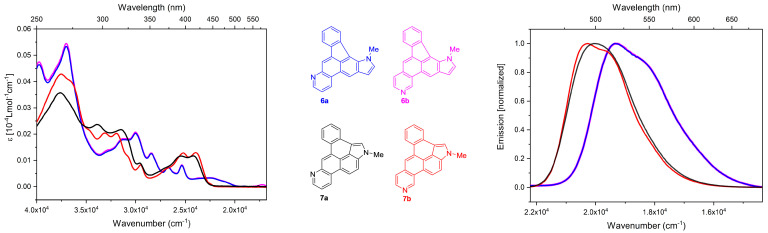
Absorption (**left**) and emission (**right**) spectra of compounds **6a,b** and **7a,b**.

Compounds **6** and **7** were studied in terms of their optical and electrochemical properties. Steady-state absorption and photoluminescence spectra were obtained in dichloromethane solution, and the determined optical properties are shown in Table 1 and Figure 2. Compounds **6** and **7** exhibit similar absorption and emission features. Similarly, both classes show distinct fine-structured absorption spectra, with specific transitions for each class of compounds. Therefore, structural alteration results in differently structured absorption spectra, whereas the orientation of the pyridinic nitrogen within the polycyclic scaffold appears to have a negligible impact on the location and structure of the absorption and emission bands. In particular, compounds **6a** and **6b** show a distinctly red-shifted emission and reduced optical quantum yields compared to compounds **7**. Interestingly, the orientation of the pyridinic nitrogen influences the quantum yields. While the quantum yield of compound **7a** was 0.48, that of **7b** was only 0.34. By contrast, no impact was detected for compounds **6**, with a determined value of 0.10 for both derivatives.

**Figure 3:**
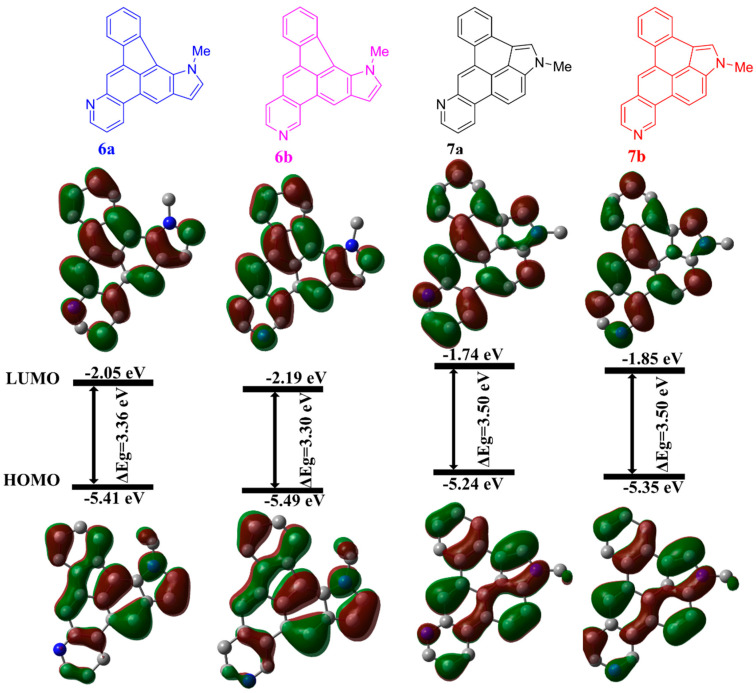
Visualization of HOMO and LUMO with associated energies.

Density Functional Theory (DFT) calculations [40] were carried out to improve our understanding of the electronic properties and aromaticity of the as-prepared compounds **6a**,**b** and **7a**,**b**. The calculated HOMO and LUMO energies are shown in Table 2 and Figure 3. The determined optical band gaps are generally smaller than the calculated values due to the exciton binding energy [41]. The electron density is distributed over the entire scaffold for all compounds. Compounds **6** have slightly reduced HOMO and LUMO, and a reduced band gap, as can be seen from their red-shifted absorption spectra compared to those of **7a** and **7b**. Compounds **6b** and **7b** have reduced HOMO and LUMO energies, possibly due to the different locations of the nitrogen atoms. Compound **6b** has a slightly reduced band gap compared to **6a.** Compounds **7a** and **7b** have the same calculated HOMO-LUMO gap, which could be explained by the nodal plane at the pyridinic nitrogen atom of **6a**.

**Figure 4:**
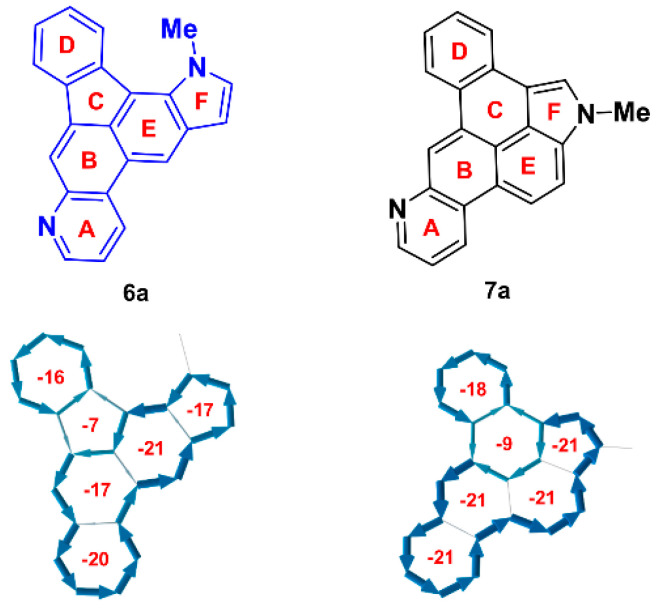
NICS(1.7)_zz_ and NICS2BC(1.25) ring currents of **6a** and **7a**.

We also calculated the nucleus-independent chemical shifts (NICSs) and performed ring-current analyses as magnetic criteria of aromaticity to evaluate the aromatic properties of compounds **6** and **7** [42,43,44,45]. NICS(1.7)_zz_ values and ring currents are almost independent of the nitrogen within the scaffold. Figure 4 exemplarily depicts the obtained NICS(1.7)_zz_ data and corresponding ring currents for compounds **6a** and **7a**.

Based on the calculated negative NICS(1.7)_zz_ values for all rings in both compounds, **6a** and **7a** can be classified as aromatic. However, the NICS(1.7)_zz_ values of the connected rings C are significantly lower. This is corroborated by NICS2BC ring-current calculations. Compounds **6a** and **7a** possess a strong global diatropic ring current along their peripheries, as well as strong semi-global ring currents in their respective indoloquinoline moieties. Local ring currents are observed for ring D in both compounds. Rings C connects both (semi-)local ring currents but form no particular diatropic ring current, which explains their reduced aromaticity.

## 4. Experimental Section

General Information: The nuclear magnetic resonance spectra (^1^H/^13^C NMR) were recorded on a Bruker AVANCE 300 III, 250 II, or 500 (Bruker, Ettlingen, Germany). The analyzed chemical shifts δ were referenced to residual solvent signals of the deuterated solvent CDCl_3_ (δ = 7.26 ppm/77.16 ppm) or DMSO-d_6_ (δ = 2.50 ppm/39.52 ppm). Infrared spectra (IR) were measured in attenuated total reflection (ATR) experiments with a Nicolet 380 FT-IR spectrometer (Thermo Fisher Scientific, Waltham, MA, USA). UV/Vis spectra were recorded on a Agilent Cary 60 UV–vis spectrophotometer, and emission spectra were recorded with an Agilent Cary Eclipse fluorescence spectrophotometer (Agilent Technologies, Santa Clara, CA, USA). Cyclovoltammetry (CV) was measured in CH_3_CN with 0.1 M *n*Bu_4_NPF_6_ as the supporting electrolyte, a glassy carbon working electrode, ANE2 (Ag/AgNO_3_ 0.01 M in CH_3_CN) as the reference electrode, and a Pt counter electrode with ferrocene as the external standard (1 mM in CH_3_CN). The potential is given vs. Fc^+^/Fc. The potentiostat used was a PalmSense EmStat3 blue (PalmSense, Houten, The Netherlands). Basic and high-resolution mass spectra (MS/HRMS) were measured on instruments paired with a preceding gas chromatograph (GC) or liquid chromatograph (LC). The samples were ionized through electron impact ionization (EI) on an Agilent 6890/5973 or Agilent 7890/5977 GC-MS equipped with an HP-5 capillary column using helium carrier gas or by applying electron spray ionization (ESI) on an Agilent 1200/6210 Time-of-Flight (TOF) LC–MS (Agilent Technologies, Santa Clara, CA, USA). The X-ray single-crystal structure analysis was performed on a Bruker Apex Kappa-II CCD diffractometer (Bruker AXS SE, Karlsruhe, Germany).

Materials: The applied solvents, 1,4-Dioxane, N-Methyl-2-pyrrolidone, DMA, and DCM, were obtained as dry solvents from commercial sources and employed without further purification. Solvents for extraction and column chromatography were available after previous distillation. If not otherwise stated, all reagents, such as 1-chloro-2-ethynylbenzene, (1-methyl-1*H*-indol-5-yl)boronic acid pinacol ester, methanesulfonic acid, catalysts, ligands, and bases, were purchased and used without further purification. Column chromatography was performed using a Merck Silica gel 60 (particle size 63–200 μm).

General Procedure for the Synthesis of 5-(2-((2-chlorophenyl)ethynyl)pyridin-3-yl)-1-methyl-1*H*-indole (**3a**) and 5-(4-((2-chlorophenyl)ethynyl)pyridin-3-yl)-1-methyl-1*H*-indole (**3b**):

To a glass pressure tube under an inert atmosphere were added **2** (250 mg, 1.0 equiv.), 2-Naphthaleneboronic acid (1.2 equiv.), Pd(PPh_3_)_4_ (5 mol%), K_3_PO_4_ (2.0 equiv.), and a mixture of 1,4-dioxane (6 mL) and H_2_O (1 mL). The reaction was heated in a metal block at 100 °C for 3 h. The solution was diluted with water and extracted with ethyl acetate three times, dried with sodium sulfate, and purified by column chromatography.

**5-(2-((2-chlorophenyl)ethynyl)pyridin-3-yl)-1-methyl-1*H*-indole (3a).** Starting with **2a** (250 mg, 0.86 mmol), **3a** was isolated as a yellow solid (183 mg, 62%); mp.: 112–114 °C. ^1^H NMR (500 MHz, Chloroform-*d*) δ 8.62 (dd, *J* = 4.7, 1.7 Hz, 1H), 7.99 (dd, *J* = 1.8, 0.7 Hz, 1H), 7.81 (dd, *J* = 7.8, 1.7 Hz, 1H), 7.54 (dd, *J* = 8.4, 1.8 Hz, 1H), 7.45 (dd, *J* = 7.7, 1.7 Hz, 1H), 7.40 (d, *J* = 8.4 Hz, 1H), 7.36–7.30 (m, 2H), 7.20 (td, *J* = 7.7, 1.8 Hz, 1H), 7.18–7.09 (m, 2H), 6.55 (dd, *J* = 3.2, 0.9 Hz, 1H), 3.83 (s, 3H). ^13^C{1H} NMR (126 MHz, Chloroform-*d*) δ 148.2, 141.5, 141.2, 137.6, 136.7, 136.4, 134.1, 129.7, 129.7, 129.3, 129.2, 128.6, 126.4, 123.3, 123.2, 122.9, 122.2, 109.2, 101.7, 94.0, 87.9, 33.1. MS (GC): *m*/*z* (%) = 345 (3), 344 (C_22_H_15_^37^ClN_2_, 13), 343 (13), 342 (M^+^, C_22_H_15_^35^ClN_2_, 38), 341 (11), 309 (3), 308 (27), 307 (100), 306 (11), 305 (8), 304 (2). HRMS (ESI-TOF) *m*/*z*: [M+H]^+^ calcd for C_22_H_15_^35^ClN_2_, 343.1002; found, 343.0999. IR (ATR): ṽ = 1618 (w), 1552 (w), 1475 (m), 1438 (m), 1416 (s), 1339 (m), 1249 (m), 1156 (w), 1076 (m), 1053 (m).

**5-(4-((2-chlorophenyl)ethynyl)pyridin-3-yl)-1-methyl-1*H*-indole (3b).** Starting with **2b** (250 mg, 0.86 mmol), **3b** was isolated as a yellow solid (159 mg, 54%); mp.: 84–86 °C. ^1^H NMR (500 MHz, Chloroform-*d*) δ 8.69 (s, 1H), 8.45 (d, *J* = 5.1 Hz, 1H), 7.91 (d, *J* = 1.0 Hz, 1H), 7.47 (dd, *J* = 8.5, 1.7 Hz, 1H), 7.44 (d, *J* = 5.1 Hz, 1H), 7.32 (d, *J* = 8.4 Hz, 1H), 7.28 (dd, *J* = 3.9, 1.5 Hz, 1H), 7.26 (dd, *J* = 4.3, 1.5 Hz, 1H), 7.17–7.12 (m, 1H), 7.07 (td, *J* = 7.6, 1.2 Hz, 1H), 7.01 (d, *J* = 3.1 Hz, 1H), 6.46 (dd, *J* = 3.0, 0.9 Hz, 1H), 3.74 (s, 3H). ^13^C{1H} NMR (126 MHz, Chloroform-*d*) δ 150.9, 147.2, 139.3, 136.7, 136.3, 133.8, 130.0, 129.7, 129.4, 129.1, 128.6, 128.0, 126.7, 126.5, 123.3, 122.7, 122.2, 109.3, 101.7, 92.7, 92.2, 33.1. MS (GC): *m*/*z* (%) = 345 (6), 344 (C_22_H_15_^37^ClN_2_, 27), 343(22), 342 (M^+^, C_22_H_15_^35^ClN_2_, 76), 341 (10), 340 (3), 309 (2), 308 (26), 307 (100), 306 (26), 305 (15), 304 (4). HRMS (ESI-TOF) *m*/*z*: [M+H]^+^ calcd for C_22_H_15_^35^ClN_2_, 343.1002; found, 343.0995. IR (ATR): ṽ = 1613 (w), 1578 (m), 1510 (m), 1484 (m), 1442 (m), 1397 (m), 1339 (m), 1245 (m), 1080 (m), 1055 (m).

General Procedure for the Synthesis of 6-(2-chlorophenyl)-8-methyl-8*H*-indolo[*6*,*5-h*]isoquinoline (**4**) and 4-(2-chlorophenyl)-1-methyl-1*H*-indolo[*4*,*5-h*]isoquinoline (**5**):

To a glass pressure tube under an inert atmosphere were added substances **3** (350 mg, 1 equiv.) and MsOH (30 equiv.). The reaction was carried out in a pressure tube and heated in a metal block at 120 °C for 18 h. Then, the cooled solution was diluted with ethyl acetate and extracted with saturated sodium bicarbonate 3 times, dried with sodium sulfate, and purified on a column.

**6-(2-chlorophenyl)-8-methyl-8*H*-indolo[5,6-*f*]quinoline (4a).** Starting with **3a** (350 mg, 1.02 mmol), **4a** was isolated as a yellow solid (38 mg, 11%); mp.: 198–200 °C. ^1^H NMR (250 MHz, Chloroform-*d*) δ 9.10–8.99 (m, 1H), 8.97 (s, 1H), 8.88 (dd, *J* = 4.4, 1.6 Hz, 1H), 7.74 (s, 1H), 7.62–7.41 (m, 5H), 7.33 (s, 1H), 7.24 (d, *J* = 3.6 Hz, 1H), 6.71 (dd, *J* = 3.2, 0.9 Hz, 1H), 3.73 (s, 3H). ^13^C{1H} NMR (63 MHz, Chloroform-*d*) δ 148.7, 147.3, 140.8, 139.7, 137.5, 134.2, 132.8, 132.1, 130.3, 129.9, 129.6, 129.3, 127.4, 127.0, 126.8, 126.7, 123.5, 121.4, 114.7, 105.4, 100.9, 33.2. MS (GC): *m*/*z* (%) = 345 (8), 344 (C_22_H_15_^37^ClN_2_, 34), 343 (26), 342 (M+, C_22_H_15_^35^ClN_2_, 100), 341 (2), 309 (2), 308 (17), 307 (72), 306 (11), 305 (14), 304 (3). HRMS (ESI-TOF) *m*/*z*: [M+H]^+^ calcd for C_22_H_15_^35^ClN_2_, 343.1002; found, 343.1000. IR (ATR): ṽ = 2920 (m), 1737 (w), 1620 (w), 1510 (m), 1461 (m), 1414 (m), 1374 (m), 1265 (m), 1082 (m), 1053 (m), 1032 (m).

**6-(2-chlorophenyl)-8-methyl-8*H*-indolo[6,5-*h*]isoquinoline (4b).** Starting with **3b** (350 mg, 1.02 mmol), **4b** was isolated as a yellow solid (109 mg, 31%); mp.: 103–105 °C. ^1^H NMR (300 MHz, Chloroform-*d*) δ 10.16 (s, 1H), 9.16 (s, 1H), 8.68 (s, 1H), 7.67 (d, *J* = 5.2 Hz, 1H), 7.64–7.59 (m, 1H), 7.51–7.43 (m, 4H), 7.35 (s, 1H), 7.30 (d, *J* = 3.2 Hz, 1H), 6.77 (dd, *J* = 3.2, 0.9 Hz, 1H), 3.75 (s, 3H). ^13^C{1H} NMR (63 MHz, Chloroform-*d*) δ 146.2, 144.6, 141.6, 139.6, 137.4, 134.6, 134.1, 133.1, 132.0, 130.0, 129.9, 129.5, 127.3, 127.0, 126.2, 124.1, 123.0, 121.3, 113.9, 105.7, 101.2, 33.2. MS (GC): *m*/*z* (%) = 345 (8), 344 (C_22_H_15_^37^ClN_2_, 33), 343 (24), 342 (M+, C_22_H_15_^35^ClN_2_, 100), 309 (2), 308 (18), 307 (74), 306 (9), 305 (11), 304 (3). HRMS (ESI-TOF) *m*/*z*: [M+H]^+^ calcd for C_22_H_15_^35^ClN_2_, 343.1002; found, 343.0997. IR (ATR): ṽ = 2922 (m), 1504 (m), 1461 (m), 1424 (s), 1414 (m), 1381 (m), 1337 (m), 1232 (s), 1053 (s), 1030 (m).

**4-(2-chlorophenyl)-1-methyl-1*H*-indolo[5,4-*f*]quinoline (5a).** Starting with **3a** (350 mg, 1.02 mmol), **5a** was isolated as a yellow solid (195 mg, 56%); mp.: 176–178 °C. ^1^H NMR (250 MHz, Chloroform-*d*) δ 9.04 (d, *J* = 9.2 Hz, 1H), 8.96 (dd, *J* = 4.3, 1.5 Hz, 1H), 8.56 (d, *J* = 9.1 Hz, 1H), 7.98 (d, *J* = 0.8 Hz, 1H), 7.70 (dd, *J* = 9.0, 0.9 Hz, 1H), 7.64–7.58 (m, 1H), 7.58–7.53 (m, 1H), 7.52–7.41 (m, 3H), 6.91 (d, *J* = 3.2 Hz, 1H), 5.35 (dd, *J* = 3.2, 0.9 Hz, 1H), 3.85 (s, 3H). ^13^C{1H} NMR (63 MHz, Chloroform-*d*) δ 149.0, 146.4, 141.8, 139.5, 135.4, 134.4, 131.5, 131.0, 129.8, 129.5, 129.2, 127.8, 127.2, 126.3, 125.3, 124.4, 124.0, 121.4, 117.2, 111.3, 102.7, 33.2. MS (GC): *m*/*z* (%) = 345 (3), 344 (C_22_H_15_^37^ClN_2_, 11), 343 (8), 342 (M^+^, C_22_H_15_^35^ClN_2_, 32), 341 (1), 309 (3), 308 (24), 307 (100), 306 (30), 305 (7), 304 (1). HRMS (ESI-TOF) *m*/*z*: [M+H]^+^ calcd for C_22_H_15_^35^ClN_2_, 343.1002; found, 343.1006. IR (ATR): ṽ = 2922 (w), 1576 (w), 1504 (w), 1469 (m), 1414 (m), 1346 (m), 1317 (m), 1261 (m), 1053 (m), 1032 (m).

**4-(2-chlorophenyl)-1-methyl-1*H*-indolo[4,5-*h*]isoquinoline (5b).** Starting with **3b** (350 mg, 1.02 mmol), **5b** was isolated as a yellow solid (168 mg, 48%); mp.: 180–182 °C. ^1^H NMR (250 MHz, Chloroform-*d*) δ 10.18 (s, 2H), 8.78 (d, *J* = 9.1 Hz, 1H), 8.68 (d, *J* = 5.4 Hz, 1H), 7.80–7.70 (m, 1H), 7.67–7.59 (m, 2H), 7.56–7.42 (m, 3H), 6.92 (d, *J* = 3.2 Hz, 1H), 5.33 (dd, *J* = 3.2, 0.9 Hz, 1H), 3.87 (s, 3H). ^13^C{1H} NMR (63 MHz, Chloroform-*d*) δ 147.3, 143.6, 141.7, 140.4, 135.4, 134.2, 133.7, 131.4, 129.8, 129.6, 127.9, 127.3, 126.1, 126.0, 125.9, 124.3, 124.1, 121.1, 116.4, 112.0, 102.6, 33.3. MS (GC): *m*/*z* (%) = 345 (2), 344 (C_22_H_15_^37^ClN_2_, 10), 343 (8), 342 (M^+^, C_22_H_15_^35^ClN_2_, 27), 341 (2), 309 (2), 308 (22), 307 (100), 306 (17), 305 (6), 304 (1). HRMS (ESI-TOF) *m*/*z*: [M+H]^+^ calcd for C_22_H_15_^35^ClN_2_, 343.1002; found, 343.1004. IR (ATR): ṽ = 1590 (vw), 1504 (w), 1414 (w), 1348 (w), 1304 (m), 1228 (vs), 1181 (s), 1121 (s), 1059 (m), 981 (s).

General Procedure for the Synthesis of **6** and **7**:

To a pressure tube under an inert atmosphere were added **4** and **5** (53–120 mg, 1.0 equiv.), PdCl_2_ (0.2 equiv.), PCy_3_ (0.4 equiv.), DBU (3.0 equiv.), and NMP as a solvent. The reaction was carried out in a pressure tube and heated in a metal block at 150 °C for 18 h. The solution was diluted with distilled water and extracted three times with dichloromethane, then extracted five or six times with distilled water to remove the NMP. The organic layer was dried with sodium sulfate, filtered, and concentrated in vacuo. The residue was purified by column chromatography (heptane/EtOAc).

**1-methyl-1*H*-pyrrolo[3′,2′:3,4]fluoreno[1,9-*fg*]quinoline (6a).** Starting with **4a** (53 mg, 0.15 mmol), **6a** was isolated as a yellow solid (38 mg, 80%); mp.: 230–232 °C. ^1^H NMR (500 MHz, DMSO-*d*_6_) δ 9.28 (d, *J* = 8.2 Hz, 1H), 8.98 (dd, *J* = 15.2, 3.3 Hz, 2H), 8.61 (s, 1H), 8.53 (d, *J* = 7.8 Hz, 1H), 8.34 (d, *J* = 7.4 Hz, 1H), 7.72 (dd, *J* = 8.3, 4.3 Hz, 1H), 7.66 (d, *J* = 2.6 Hz, 1H), 7.51 (t, *J* = 7.5 Hz, 1H), 7.44 (t, *J* = 7.3 Hz, 1H), 6.88–6.83 (m, 1H), 4.49 (s, 3H). ^13^C{1H} NMR (126 MHz, DMSO-*d*_6_) δ 148.7, 148.2, 139.0, 137.7, 137.3, 135.2, 134.4, 133.5, 130.9, 128.9, 127.0, 126.0, 123.8, 122.7, 121.6, 120.6, 120.0, 117.5, 115.5, 102.5, 38.6. MS (GC): *m*/*z* (%) = 308 (3), 307 (24), 306 (M^+^, C_22_H_14_N_2_, 100), 305 (25), 304 (4), 303 (3). HRMS (ESI-TOF) m/z: [M+H]^+^ calcd for C_22_H_15_N_2_, 307.1235; found, 307.1232. IR (ATR): ṽ = 2920 (w), 1583 (w), 1527 (w), 1426 (m), 1401 (m), 1350 (m), 1315 (m), 1259 (s), 1080 (s), 1016 (s).

**1-methyl-1*H*-pyrrolo[3′,2′:3,4]fluoreno[9,1-*gh*]isoquinoline (6b).** Starting with **4b** (73 mg, 0.21 mmol), **6b** was isolated as a yellow solid (57 mg, 87%); mp.: 207–209 °C. ^1^H NMR (300 MHz, DMSO-*d*_6_) δ 10.15 (s, 1H), 9.03 (s, 1H), 8.70 (d, *J* = 5.4 Hz, 1H), 8.48–8.41 (m, 2H), 8.19 (dd, *J* = 7.2, 0.9 Hz, 1H), 8.00 (d, *J* = 4.7 Hz, 1H), 7.64 (d, *J* = 3.2 Hz, 1H), 7.45 (dtd, *J* = 22.7, 7.4, 1.2 Hz, 2H), 6.82 (d, *J* = 3.2 Hz, 1H), 4.43 (s, 3H). ^13^C{1H} NMR (75 MHz, DMSO-*d*_6_) δ 146.5, 144.4, 139.4, 138.1, 137.0, 135.6, 135.3, 134.7, 133.3, 129.1, 127.6, 126.0, 124.8, 123.8, 122.5, 122.3, 119.4, 117.7, 117.4, 114.8, 102.6, 39.2. MS (GC): *m*/*z* (%) = 308 (3), 307 (24), 306 (M^+^, C_22_H_14_N_2_, 100), 305 (27), 304 (6), 303 (2). HRMS (ESI-TOF) *m*/*z*: [M+H]^+^ calcd for C_22_H_15_N_2_, 307.1235; found, 307.1231. IR (ATR): ṽ = 2922 (m), 1595 (m), 1529 (m), 1447 (m), 1352 (m), 1317 (m), 1259 (m), 1199 (m), 1086 (s), 1016 (s).

**7-methyl-7*H*-pyrrolo[2′,3′,4′:4,10]anthra[1,9-*fg*]quinoline (7a).** Starting with **5a** (120 mg, 0.35 mmol), **7a** was isolated as a yellow solid (102 mg, 95%); mp.: 198–200 °C. ^1^H NMR (500 MHz, DMSO-*d*_6_) δ 9.29 (d, *J* = 6.9 Hz, 1H), 9.05 (dd, *J* = 4.1, 1.6 Hz, 1H), 9.00 (s, 1H), 8.85 (d, *J* = 7.9 Hz, 1H), 8.71 (d, *J* = 8.9 Hz, 1H), 8.22 (d, *J* = 11.9 Hz, 2H), 8.06 (d, *J* = 8.8 Hz, 1H), 7.71 (dd, *J* = 8.3, 4.2 Hz, 1H), 7.64 (t, *J* = 6.8 Hz, 1H), 7.58–7.53 (m, 1H), 4.19 (s, 3H). ^13^C{1H} NMR (126 MHz, DMSO-*d*_6_) δ 148.9, 146.6, 131.1, 130.8, 130.5, 129.6, 129.0, 128.7, 125.7, 125.5, 124.4, 123.7, 122.5, 122.2, 121.0, 120.9, 120.7, 119.3, 117.3, 112.3, 112.1, 33.7. MS (GC): *m*/*z* (%) = 308 (3), 307 (29), 306 (M^+^, C_22_H_14_N_2_, 100), 305 (16), 304 (3), 303 (2). HRMS (ESI-TOF) *m*/*z*: [M+H]^+^ calcd for C_22_H_15_N_2_, 307.1235; found, 307.1233. IR (ATR): ṽ = 1607 (w), 1529 (w), 1475 (w), 1418 (w), 1374 (m), 1350 (m), 1282 (w), 1230 (m), 1170 (w), 1043 (m).

**1-methyl-1*H*-pyrrolo[2′,3′,4′:4,10]anthra[9,1-*gh*]isoquinoline (7b).** Starting with **5b** (95 mg, 0.28 mmol), **7b** was isolated as a yellow solid (84 mg, 99%); mp.: 232–234 °C. ^1^H NMR (300 MHz, Chloroform-*d*) δ 10.01 (s, 1H), 8.67 (d, *J* = 5.5 Hz, 1H), 8.50 (dd, *J* = 8.1, 1.4 Hz, 1H), 8.44 (d, *J* = 8.9 Hz, 1H), 8.36 (s, 1H), 7.94 (dd, *J* = 7.8, 1.4 Hz, 1H), 7.83 (dd, *J* = 5.6, 0.9 Hz, 1H), 7.59–7.43 (m, 4H), 3.98 (s, 3H). ^13^C{1H} NMR (75 MHz, Chloroform-*d*) δ 146.6, 142.7, 134.4, 132.3, 130.8, 130.0, 129.4, 128.6, 125.5, 125.2, 124.2, 123.4, 122.6, 122.3, 122.1, 121.5, 120.3, 116.2, 116.2, 113.1, 111.4, 33.9. MS (GC): *m*/*z* (%) = 308 (3), 307 (20), 306 (M^+^, C_22_H_14_N_2_, 100), 305 (15), 304 (3), 303 (2). HRMS (ESI-TOF) *m*/*z*: [M+H]^+^ calcd for C_22_H_15_N_2_, 307.1235; found, 307.1237. IR (ATR): ṽ = 1607 (m), 1531 (m), 1453 (m), 1412 (m), 1372 (m), 1350 (m), 1224 (s), 1148 (m), 1039 (m).

## 5. Conclusions

Two isomers of each pyrrolo[3′,2′:3,4]fluoreno[9,1-*gh*]quinoline and pyrrolo [2′,3′,4′:4,10]anthra[1,9-*fg*]quinoline derivative were synthesized via cycloisomerization and Pd-catalyzed CH arylation with excellent yield. Despite the fluorescence quantum yields, the location of the nitrogen atom had a negligible impact on the optical and aromatic properties. However, altering the size of the central ring led to changes in the optical and aromatic properties of the polycyclic scaffold, highlighting the potential of bottom-up synthesis for the fine-tuning of respective properties.

## Figures and Tables

**Table 1:** Optical data of **6a,b** and **7a,b**.

	6a	6b	7a	7b
*λ_abs1_* [nm]	446	446	414	418
(*ε*_1_ [a])	0.15	0.15	0.28	0.31
*λ_abs2_* [nm]	395	395	392	397
(*ε*_2_ [a])	0.22	0.22	0.28	0.30
*λ_abs3_* [nm]	374	374	339	393
(*ε*_3_ [a])	0.21	0.21	0.23	0.22
*λ_em1_* [nm]	519	519	500	494
*λ_em2_* [nm]	541 ^sh^	541 ^sh^	-	509 ^sh^
*φ* [%] [b]	10	10	48	34

[a] [10^–4^ L mol^–1^ cm^–1^]; [b] fluorescence standard: coumarine 153 in EtOH (ϕ = 0.38) [39]; ^sh^ shoulder.

**Table 2:** Optical and DFT-calculated data for compounds **6** and **7**.

	UV/vis	DFT		
	E_g_^opt.^ (eV) ^(a)^	HOMO^DFT^ (eV) ^(b)^	LUMO^DFT^ (eV) ^(b)^	ΔE_g_^DFT^ (eV)
6a	2.62	−5.41	−2.05	3.36
6b	2.62	−5.49	−2.19	3.30
7a	2.90	−5.24	−1.74	3.50
7b	2.90	−5.35	−1.85	3.50

^(a)^ Estimated from the intersection of normalized absorption and emission spectra. ^(b)^ Calculated at the B3LYP/6-311G(d, p) level of theory.

## Data Availability

The data underlying this study are available in the published article and its supporting information.

## Supplementary Materials

The following supporting information can be downloaded at: https://www.mdpi.com/article/10.3390/molecules30122615/s1, Figure S1: ORTEPs of **4b**, **5b** and **6a**; Figures S2–S27: NMR Spectra Table S1: Single crystal X-ray diffraction data; Tables S2–S5 Cartesian coordinates of the optimized ground-state (S0) structure **6a**–**7b**.
