# Supplementary material for: Synthesis and Properties of 1H-Pyrrolo[3′,2′:3,4]fluoreno[9,1-gh]quinolines and 7H-Pyrrolo[2′,3′,4′:4,10]anthra[1,9-fg]quinolines
Source: Molecules. 2025 Jun 16;30(12):2615. doi: 10.3390/molecules30122615 (PMC12195833; doi:10.3390/molecules30122615)
Supplement: Supplementary file 1 [file molecules-30-02615-s001.zip › molecules-3636937-supplementary.pdf]

SUPPORTING INFORMATION

Synthesis and Properties of 1*H*-Pyrrolo[3',2':3,4]fluoreno[9,1-*gh*]quinolines and 7*H*-Pyrrolo[2',3',4':4,10]anthra[1,9-*fg*]quinolines

Aleksandra Khomutetckaia[a], Peter Ehlers[a] *, Alexander Villinger[a], Peter Langer[a,b] *

[a] Universität Rostock, Institut für Chemie, A.-Einstein-Str. 3a, 18059 Rostock, Germany

Tel.: +49 381 498 6410, Fax: +49 381 498 6412.

[b] Leibniz Institut für Katalyse an der Universität Rostock, A.-Einstein-Str.29a, 18059 Rostock, Germany

*Corresponding authors: peter.ehlers@uni-rostock.de & peter.langer@uni-rostock.de

Table of Contents

Single-crystal X-ray diffraction data 2

DFT Calculation 4

NMR-Spectra 12

Single-crystal X-ray diffraction data

Crystals of **4b** and **5b** were obtained by slow evaporation of dichloromethane/heptane at 20°C. Crystals of **6a** was obtained by slow evaporation of dichloromethane/DMSO at 20°C. X-ray single crystal structure analysis was performed on a Bruker Apex Kappa-II CCD-diffractometer.

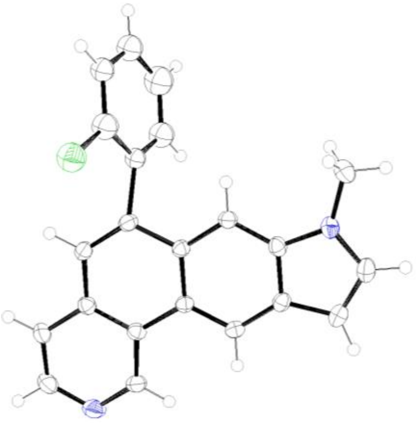

4b

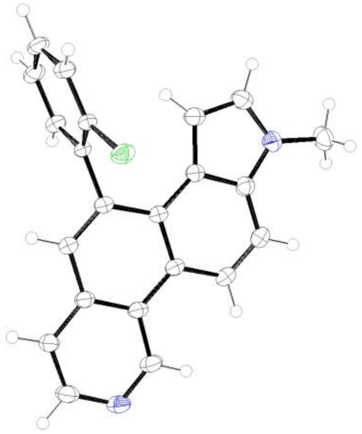

5b

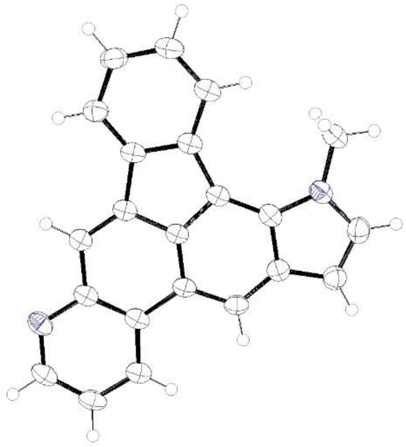

6a

Figure S1. ORTEPs of **4b**, **5b** and **6a** (determined from X-ray structural analysis, probability of ellipsoids is 50%).

Table S1. Single crystal X-ray diffraction data

	**4b**	**5b**	**6a**
**Chem. Formula**	$C_{22}H_{15}ClN_2$	$C_{22}H_{15}ClN_2$	$C_{22}H_{14}N_2$
**Form. Weight [g mol^{-1}]**	342.81	342.81	306.35
**Cryst. system**	triclinic	triclinic	monoclinic
**Space group**	P -1	P -1	P 21/n
**(Hall group)**	-P 1	-P 1	-P 2yn
**Color**	yellow	yellow	red
**a [Å]**	7.4344 (4)	11.4369 (6)	9.6777 (8)
**b [Å]**	9.8606 (5)	12.8094 (7)	8.6313 (7)
**c [Å]**	12.0372 (7)	12.9406 (7)	17.3393(15)
**α [°]**	110.548 (2)	67.073 (2)	90
**β [°]**	92.394 (2)	68.670 (2)	91.214
**γ [°]**	99.267 (2)	79.743 (2)	90
**V [Å^3]**	810.87 (8)	1624.97 (15)	1448.0 (2)
**Z**	2	4	4
**N_{ref}**	5169	9448	3497
**θ [°]**	30.997	30.000	27.997
**h,k,l_{max}**	10, 14, 17	16, 18, 18	12, 11, 22
**Dx [g cm^{-3}]**	1.404	1.401	1.405
**μ [mm^{-1}]**	0.242	0.241	0.083
**$λ_{MoK\backslash α}$ [Å]**	0.0021	0.0021	0.0021
**T [K]**	123	123	123
**F(000)**	356.0	712.0	640.0
**Npar**	244	497	218
**R**	0.0531 (3995)	0.0497 (8060)	0.0468 (2607)
**w*R***	0.1431 (5169)	0.1412 (9448)	0.1275 (3497)
S	1.073	1.104	1.027

DFT Calculation

Calculated by DFT at the B3LYP/6-311G(p) level of theory for ground state in gas phase. The local minima of the optimized geometries were confirmed by frequency calculations, ensuring that no imaginary frequencies are presented. The stated Energy E for the S_0 state is the sum of electronic and zero-point Energies.

S_0: 6a

E= -956.523265 Hartree

Table S2. *Cartesian coordinates of the optimized ground-state (S0) structure of* ***6a***

Symbol	X	Y	Z
C	4.1683910	1.4705520	-0.0002560
H	4.3194820	0.8595270	0.8919710
H	4.3190050	0.8592850	-0.8923910
H	4.9161600	2.2630670	-0.0005660
N	-4.1759050	-1.3181600	-0.0000990
C	-1.8559630	-1.8408560	-0.0000850
H	-2.1646880	-2.8793950	-0.0001600
C	-0.7385010	2.2890030	0.0001060
H	-1.4243870	3.1277390	0.0001430
C	-1.1978200	0.9650400	0.0000540
N	2.8538870	2.0853090	-0.0000160
C	1.3530400	3.7752980	0.0000180
H	0.9316340	4.7678560	0.0000270
C	2.6754480	3.4609050	-0.0000310
H	3.5399140	4.1069720	-0.0001240

C	1.6113550	1.4715440	0.0000360
C	3.0898510	-1.7067340	0.0001560
H	3.9373110	-1.0397650	0.0002590
C	3.3281940	-3.0828130	0.0001430
H	4.3515280	-3.4416550	0.0002280
C	2.2761800	-3.9976230	0.0000300
H	2.4823780	-5.0617390	0.0000180
C	0.9591860	-3.5409790	-0.0000570
H	0.1341950	-4.2449930	-0.0001180
C	0.7140340	-2.1732520	-0.0000430
C	1.1820420	0.1354460	0.0000210
C	1.7800440	-1.2261970	0.0000520
C	-0.2216990	-0.0491240	0.0000010
C	-0.5539460	-1.4445670	-0.0000420
C	0.6311860	2.5364280	0.0001030
C	-2.9018760	-0.8534450	-0.0000530
C	-2.5899290	0.5421950	0.0000260
C	-3.6764190	1.4348230	0.0000750
H	-3.5023250	2.5043430	0.0001450
C	-4.9679970	0.9491100	0.0000370
H	-5.8192750	1.6195870	0.0000720
C	-5.1640570	-0.4414130	-0.0000570
H	-6.1712690	-0.8509390	-0.0001020

S_0: 6b

E= -956.520847 Hartree

Table S3. *Cartesian coordinates of the optimized ground-state (S0) structure of* ***6b***

Symbol	X	Y	Z
C	-4.1552390	-1.5073020	-0.0002660
H	-4.3136900	-0.8983520	0.8921310
H	-4.3131870	-0.8980910	-0.8925680
H	-4.8935670	-2.3085620	-0.0005950
C	1.8331620	1.8722280	-0.0000890
H	2.1003350	2.9238800	-0.0001710
C	0.7612320	-2.2723280	0.0001090
H	1.4555320	-3.1033790	0.0001450
C	1.2068260	-0.9430880	0.0000510
N	-2.8336100	-2.1066450	-0.0000140
C	-1.3135800	-3.7798920	0.0000230
H	-0.8806730	-4.7674120	0.0000350
C	-2.6393120	-3.4802790	-0.0000300
H	-3.4963750	-4.1361350	-0.0001220
C	-1.5982140	-1.4792240	0.0000390
C	-3.1104170	1.6816560	0.0001550
H	-3.9501680	1.0051030	0.0002600
C	-3.3641940	3.0551760	0.0001450
H	-4.3915000	3.4023750	0.0002310

C	-2.3230110	3.9818600	0.0000340
H	-2.5411610	5.0435270	0.0000230
C	-1.0009140	3.5395480	-0.0000520
H	-0.1849430	4.2541430	-0.0001120
C	-0.7404940	2.1748340	-0.0000400
C	-1.1827630	-0.1392010	0.0000210
C	-1.7956010	1.2158950	0.0000510
C	0.2189380	0.0599220	0.0000010
C	0.5356030	1.4592310	-0.0000440
C	-0.6057360	-2.5332310	0.0001090
C	2.8873760	0.8949090	-0.0000600
C	2.5923600	-0.5003620	0.0000190
C	3.6897150	-1.3864310	0.0000720
H	3.5129980	-2.4581640	0.0001360
C	5.2258940	0.3026020	-0.0000430
H	6.2767710	0.5761490	-0.0000700
C	4.2462490	1.2720830	-0.0000990
H	4.5158910	2.3227830	-0.0001670
N	4.9616740	-1.0204270	0.0000450

S_0: 7a

E= -956.538859 Hartree

Table S4. *Cartesian coordinates of the optimized ground-state (S0) structure of* ***7a***

Symbol	X	Y	Z
C	1.1750920	2.3341230	0.0000520
C	0.0394750	3.1671720	0.0002690
H	0.1163700	4.2483840	0.0003400
C	-1.2055070	2.5603100	0.0001030
H	-2.0778200	3.2019290	0.0001420
C	-1.3895640	1.1428950	-0.0000380
C	-2.6712890	0.4798220	-0.0000540
C	-3.9153350	1.1448430	-0.0000450
H	-3.9554880	2.2275130	-0.0000680
C	-5.0834640	0.4169230	-0.0000190
H	-6.0504100	0.9060470	-0.0000120
C	-5.0026570	-0.9897370	-0.0000100
H	-5.9123350	-1.5860160	0.0000410
C	-2.7018350	-0.9503860	-0.0000070
C	-1.4975260	-1.7139130	-0.0000100
H	-1.6324650	-2.7870530	0.0000590
C	-0.2598620	-1.1143360	-0.0000480
C	1.0380460	-1.8386260	0.0000670
C	1.0717560	-3.2442210	0.0000260

H	0.1416710	-3.7979840	-0.0000350
C	2.2638830	-3.9502960	0.0000510
H	2.2479380	-5.0341700	0.0000120
C	3.4811720	-3.2640780	0.0000620
H	4.4173770	-3.8109760	0.0000480
C	3.4855820	-1.8797570	0.0000660
H	4.4297150	-1.3456420	0.0000330
C	2.2894180	-1.1443360	0.0001030
C	2.2684980	0.3082440	-0.0000410
C	1.0067890	0.9495560	-0.0001390
C	-0.2363880	0.3233350	-0.0000840
C	3.1983790	1.3335870	-0.0004160
H	4.2768820	1.3127390	0.0000650
C	3.1821310	3.8582930	0.0000580
H	2.9037940	4.4307780	-0.8892680
H	2.9041580	4.4304070	0.8897370
H	4.2628340	3.7218480	-0.0001940
N	2.5395490	2.5573040	-0.0000840
N	-3.8671180	-1.6564930	0.0000020

S_0: 7b

E= -956.536437 Hartree

Table S5.** Cartesian coordinates of the optimized ground-state (S0) structure of **7b

Symbol	X	Y	Z
C	1.1524260	2.3374420	0.0000380
C	0.0079180	3.1571530	0.0002480
H	0.0726050	4.2391360	0.0003250
C	-1.2309920	2.5376190	0.0000810
H	-2.1098740	3.1691830	0.0001270
C	-1.3992450	1.1182350	-0.0000630
C	-2.6710940	0.4369170	-0.0000760
C	-3.9246650	1.0936330	-0.0000690
H	-3.9613370	2.1792540	-0.0000860
C	-5.0968080	-0.8667800	0.0000430
H	-6.0747640	-1.3385420	0.0001100
C	-2.6854910	-0.9896290	-0.0000290
C	-1.4750730	-1.7424020	-0.0000020
H	-1.5669400	-2.8214890	0.0000930
C	-0.2436810	-1.1268870	-0.0000520
C	1.0631570	-1.8359180	0.0000600
C	1.1160600	-3.2412590	0.0000130
H	0.1950010	-3.8099330	-0.0000650
C	2.3166100	-3.9325420	0.0000410

H	2.3144820	-5.0164950	-0.0000070
C	3.5250920	-3.2308730	0.0000670
H	4.4681060	-3.7658420	0.0000580
C	3.5117410	-1.8469540	0.0000750
H	4.4488790	-1.3008240	0.0000500
C	2.3063520	-1.1262960	0.0001050
C	2.2685720	0.3249570	-0.0000420
C	0.9998940	0.9511750	-0.0001540
C	-0.2364580	0.3114700	-0.0001060
C	3.1870190	1.3613070	-0.0004250
H	4.2656980	1.3524030	0.0000860
C	3.1421370	3.8851090	0.0001080
H	2.8566390	4.4538220	-0.8892170
H	2.8569800	4.4534200	0.8898010
H	4.2243350	3.7614160	-0.0001310
N	2.5147660	2.5762760	-0.0000710
C	-3.9507290	-1.6245820	0.0000350
H	-4.0101060	-2.7077580	0.0000850
N	-5.0970720	0.4875640	-0.0000150

NMR-Spectra

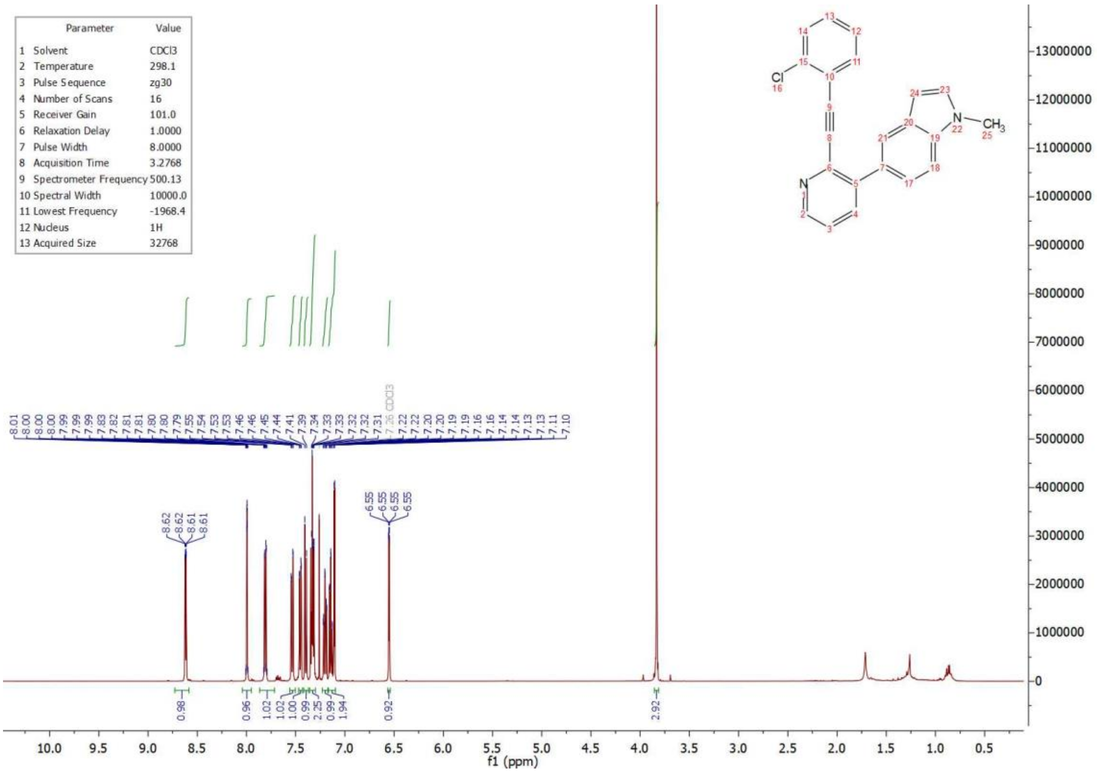

Figure S2. ^{1}H-NMR-spectrum of **3a**

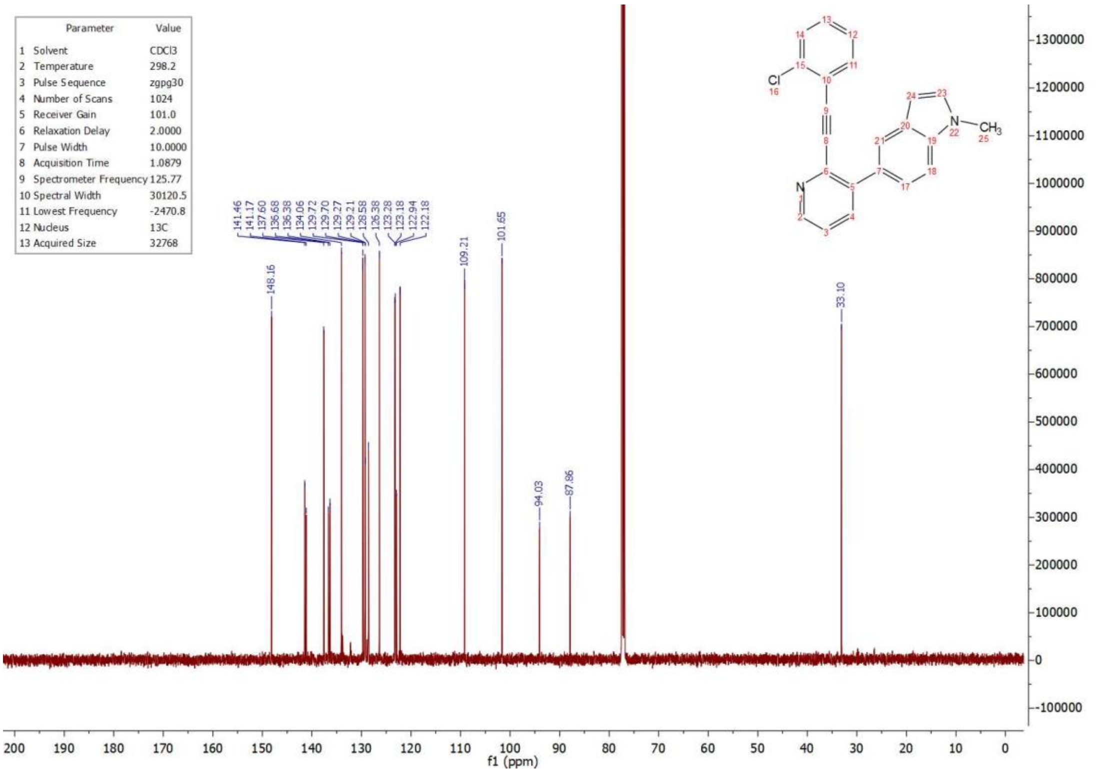

Figure S3. ^{13}C-NMR-spectrum of **3a**

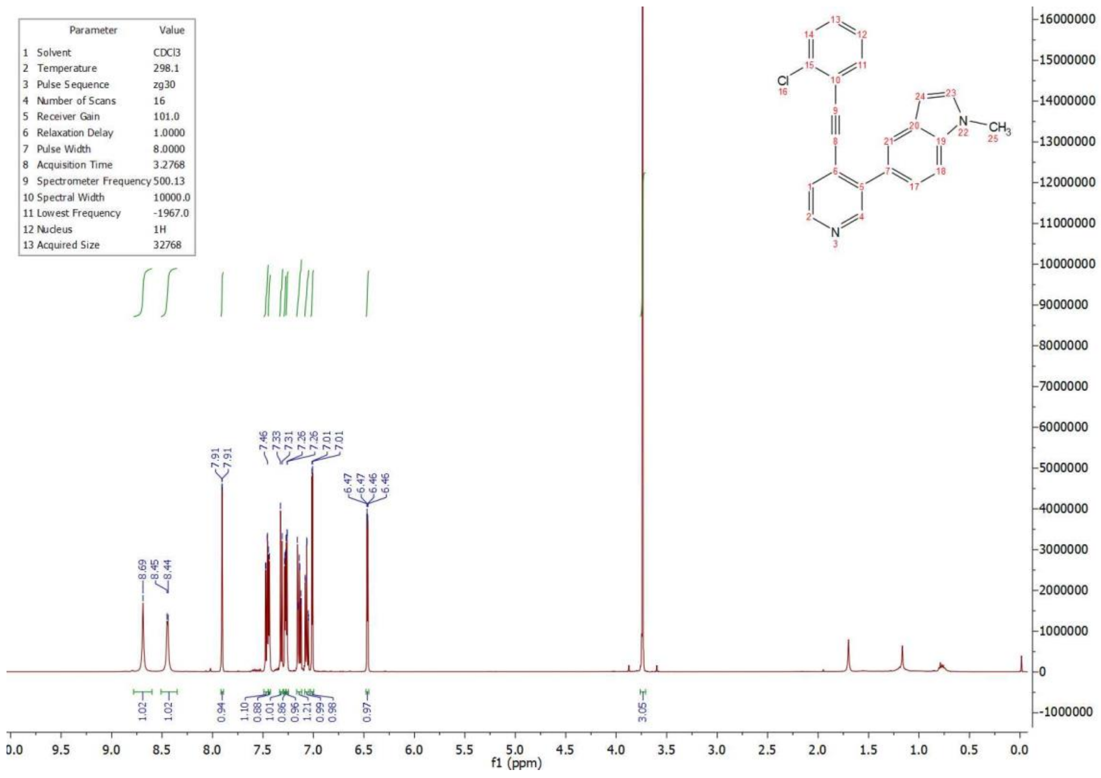

Figure S4. ^{1}H-NMR-spectrum of **3b**

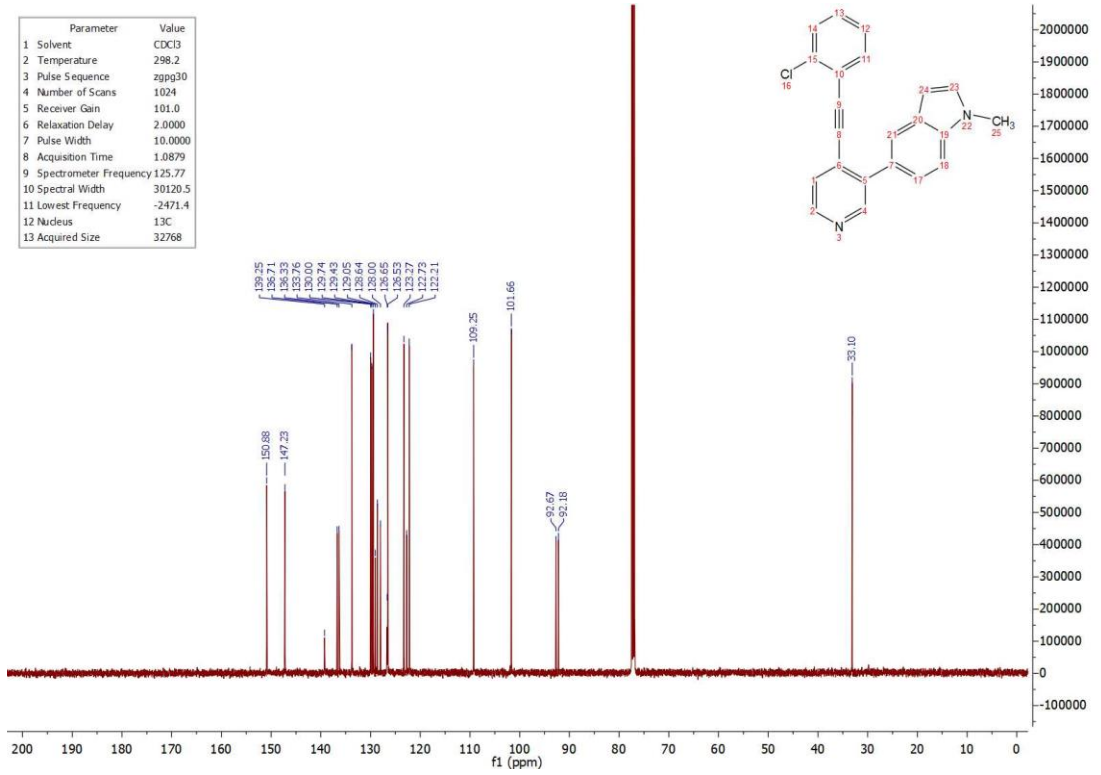

Figure S5. ^{13}C-NMR-spectrum of **3b**

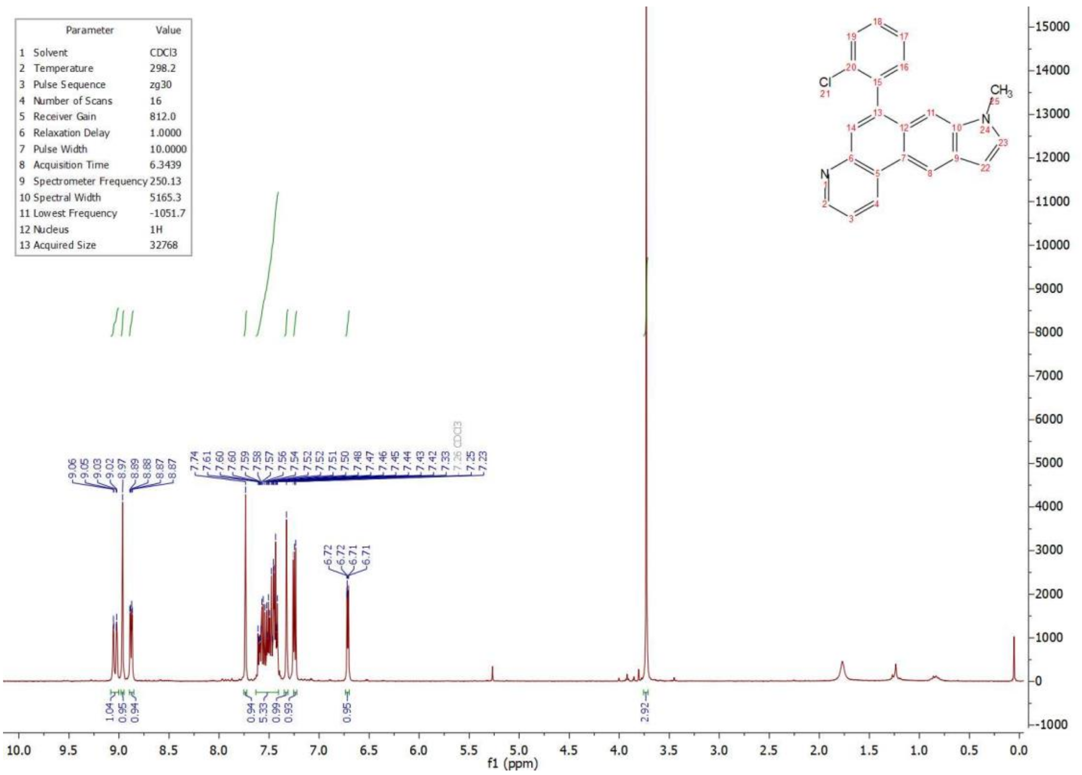

Figure S6. ^{1}H-NMR-spectrum of **4a**

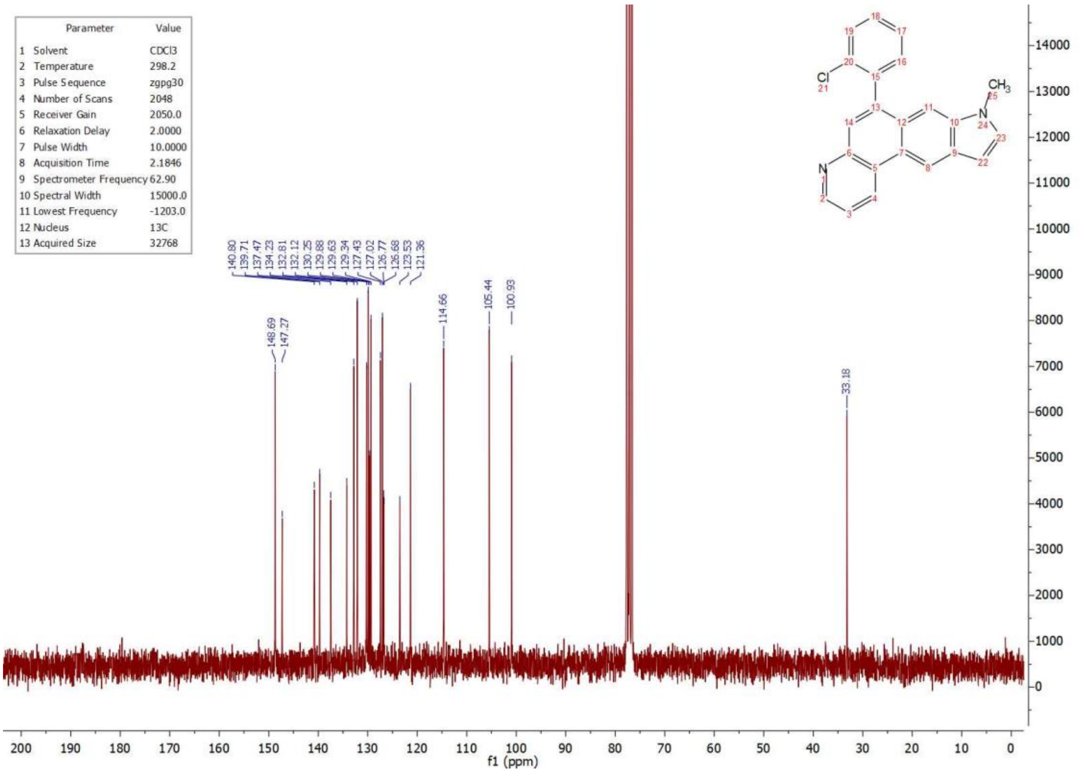

Figure S7. ^{13}C-NMR-spectrum of **4a**

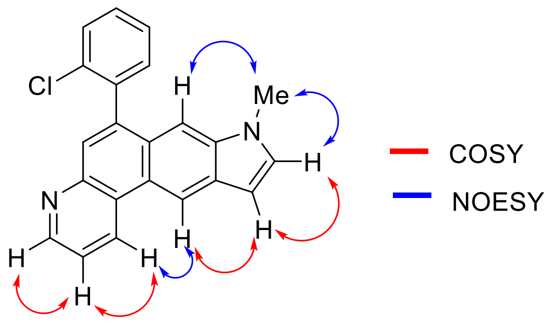

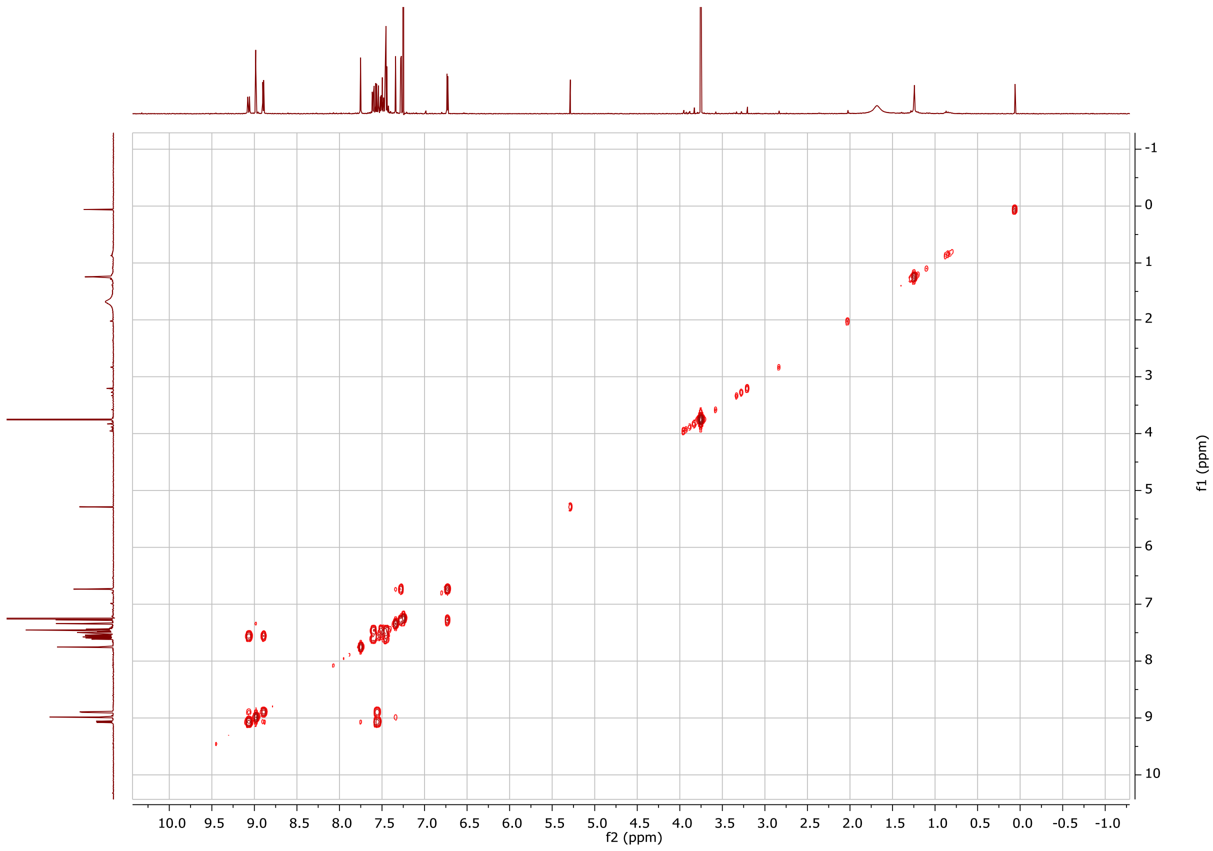

Figure S8. COSY-Spectrum of **4a**

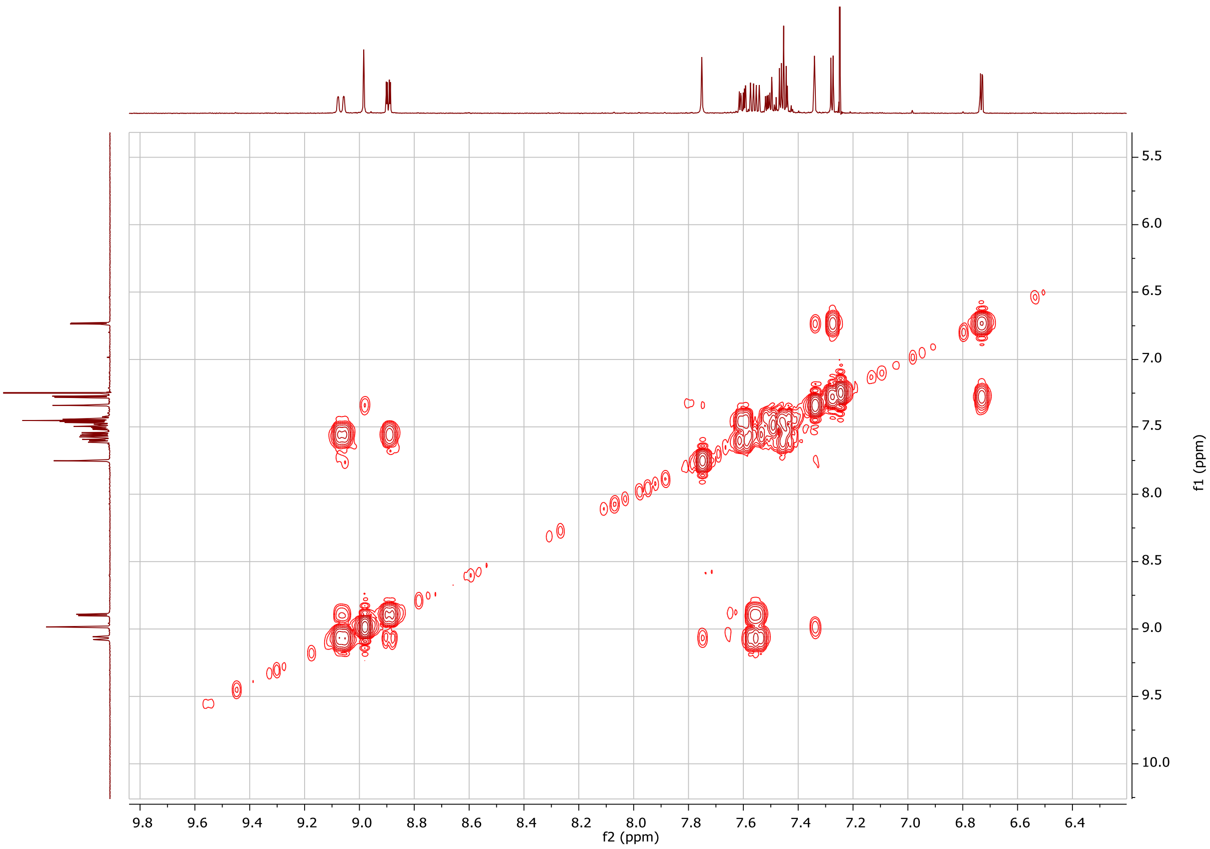

Figure S9. Enlarged COSY-Spectrum of **4a**

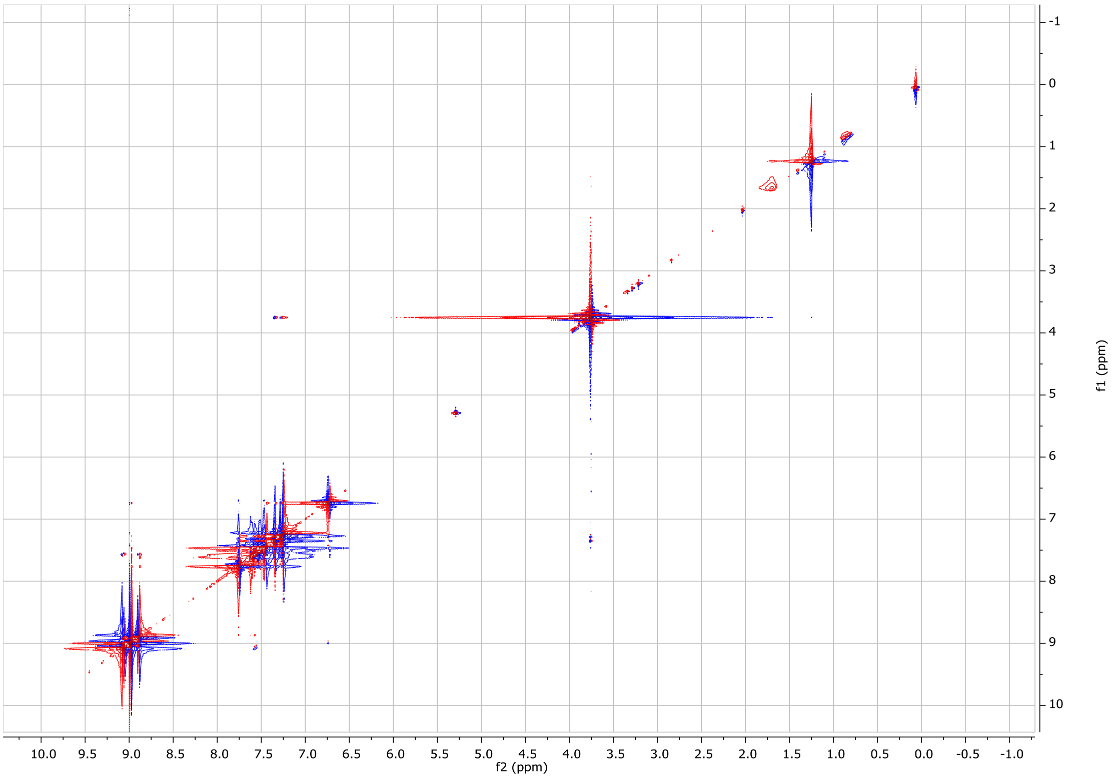

Figure S10. NOESY-Spectrum of **4a**

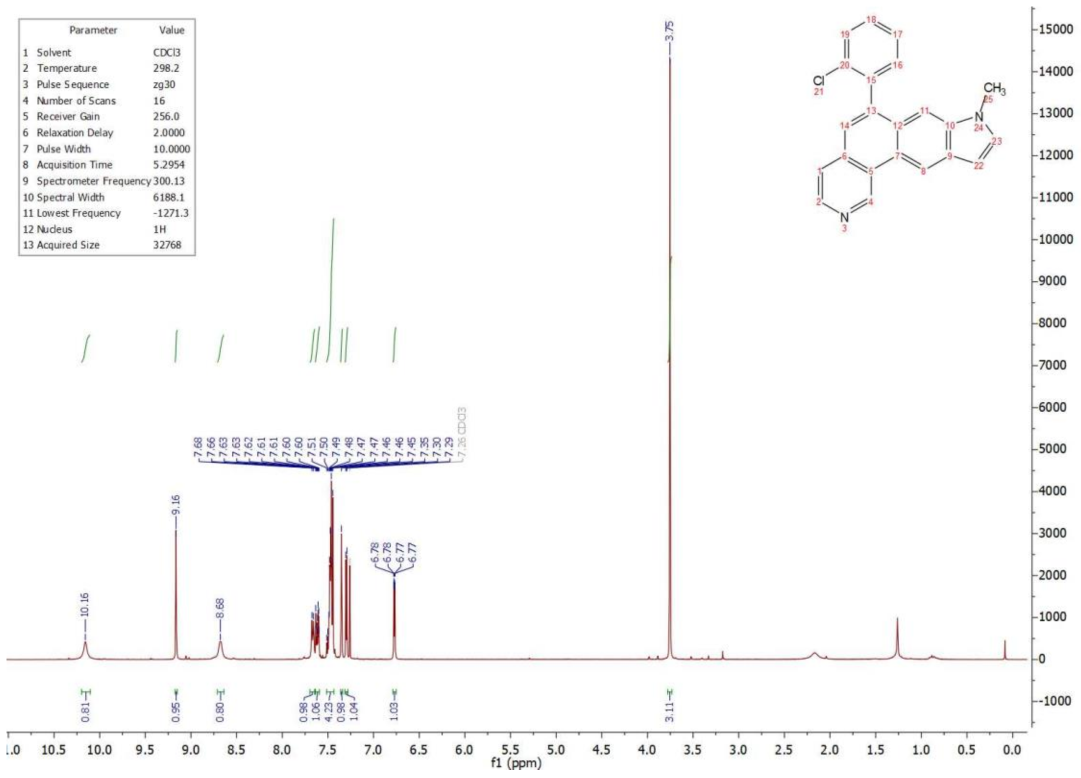

Figure S11. ^{1}H-NMR-spectrum of **4b**

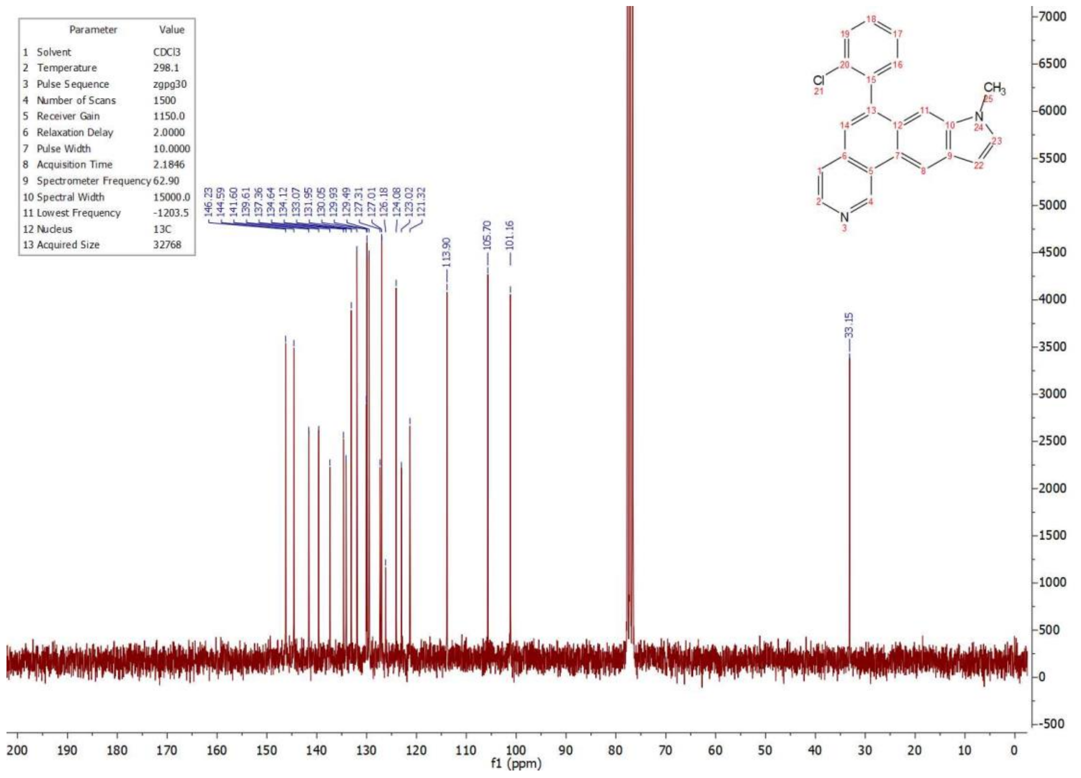

Figure S12. ^{13}C-NMR-spectrum of **4b**

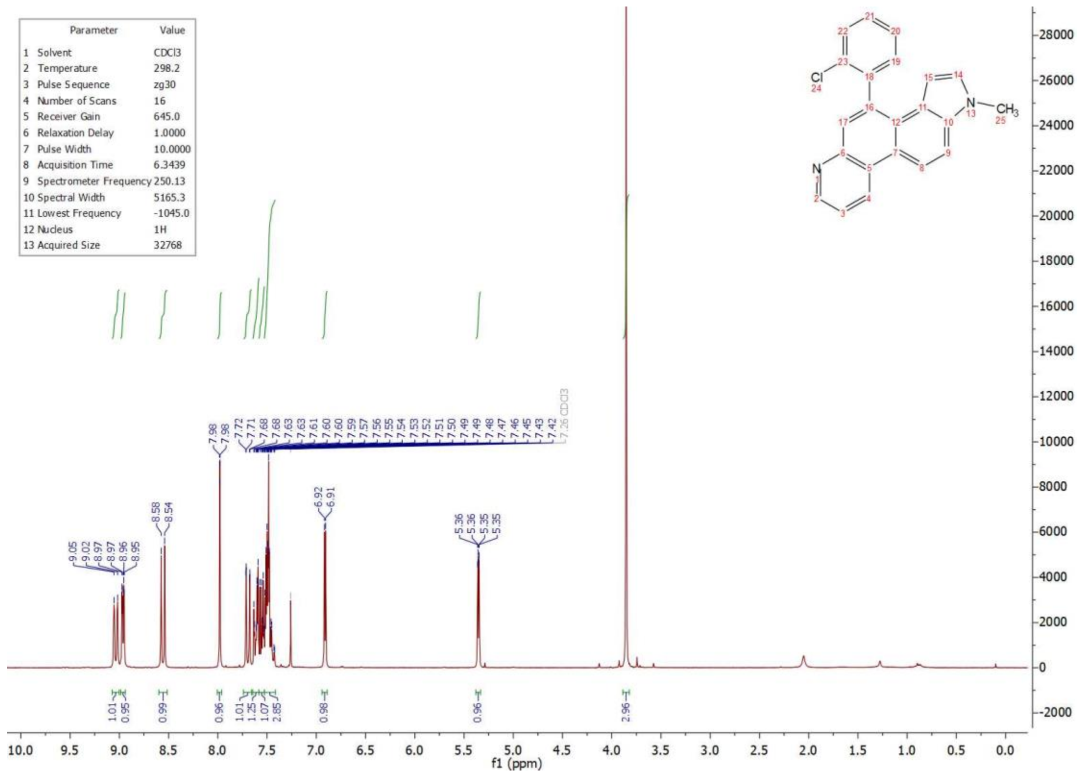

Figure S13. ^{1}H-NMR-spectrum of **5a**

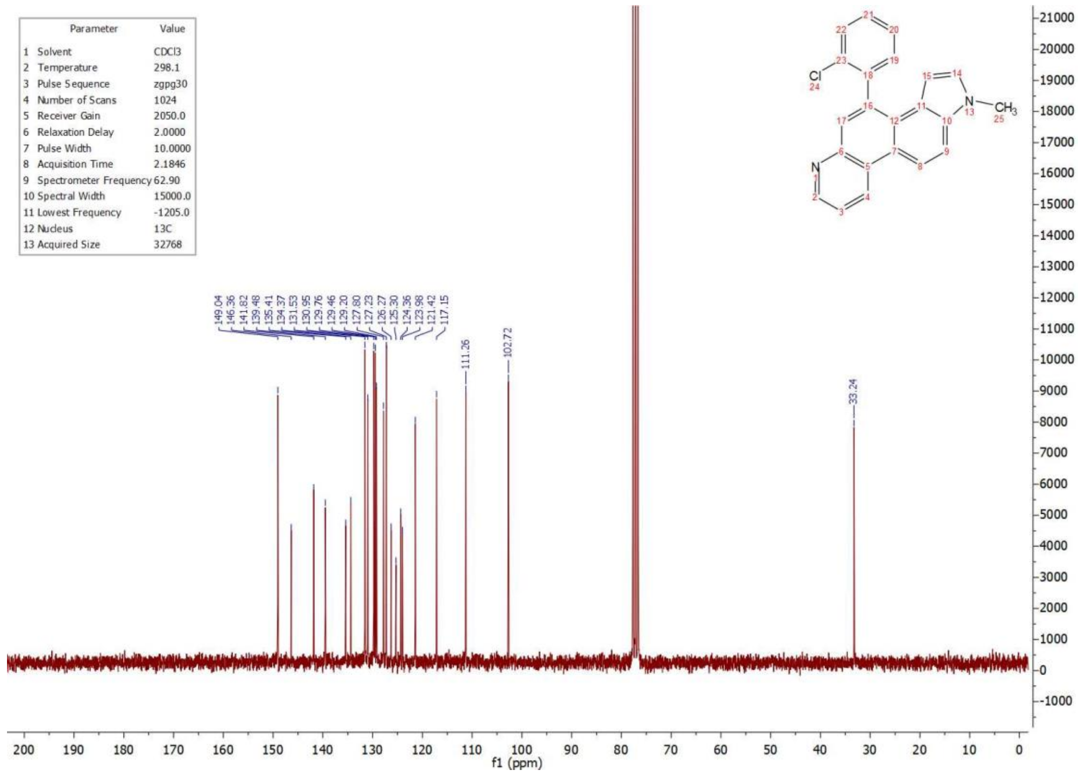

Figure S14. ^{13}C-NMR-spectrum of **5a**

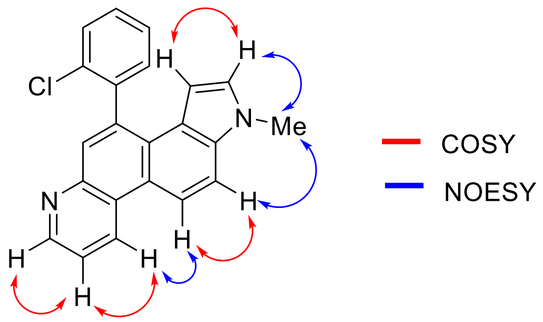

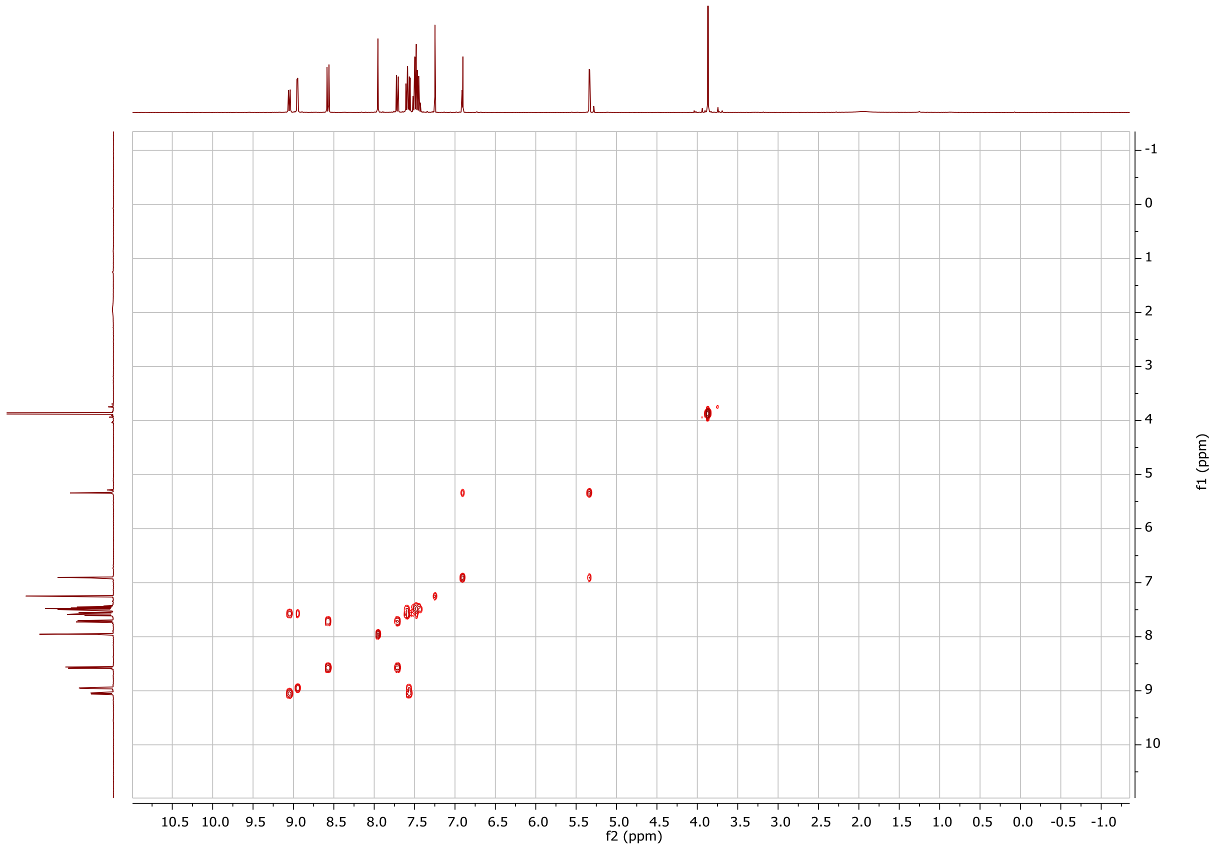

Figure S15. COSY-Spectrum of **5a**

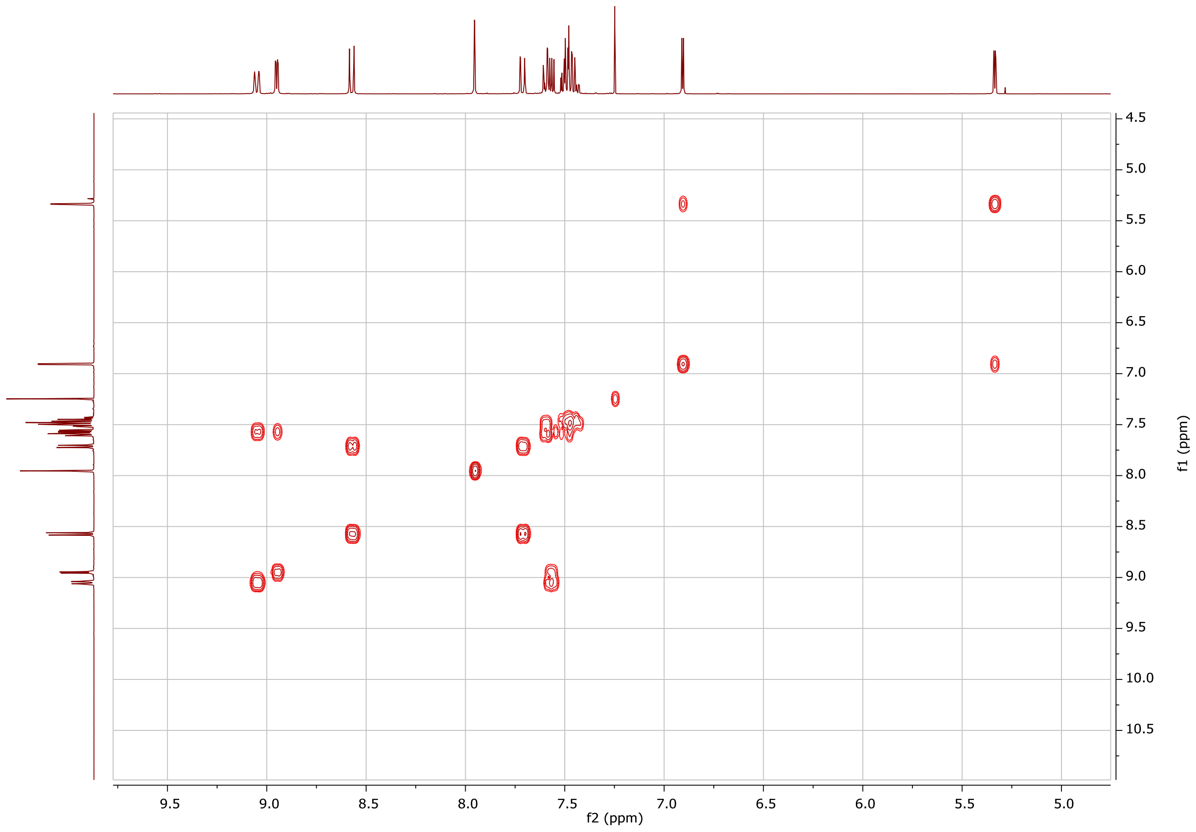

Figure S16. Enlarged COSY-Spectrum of **5a**

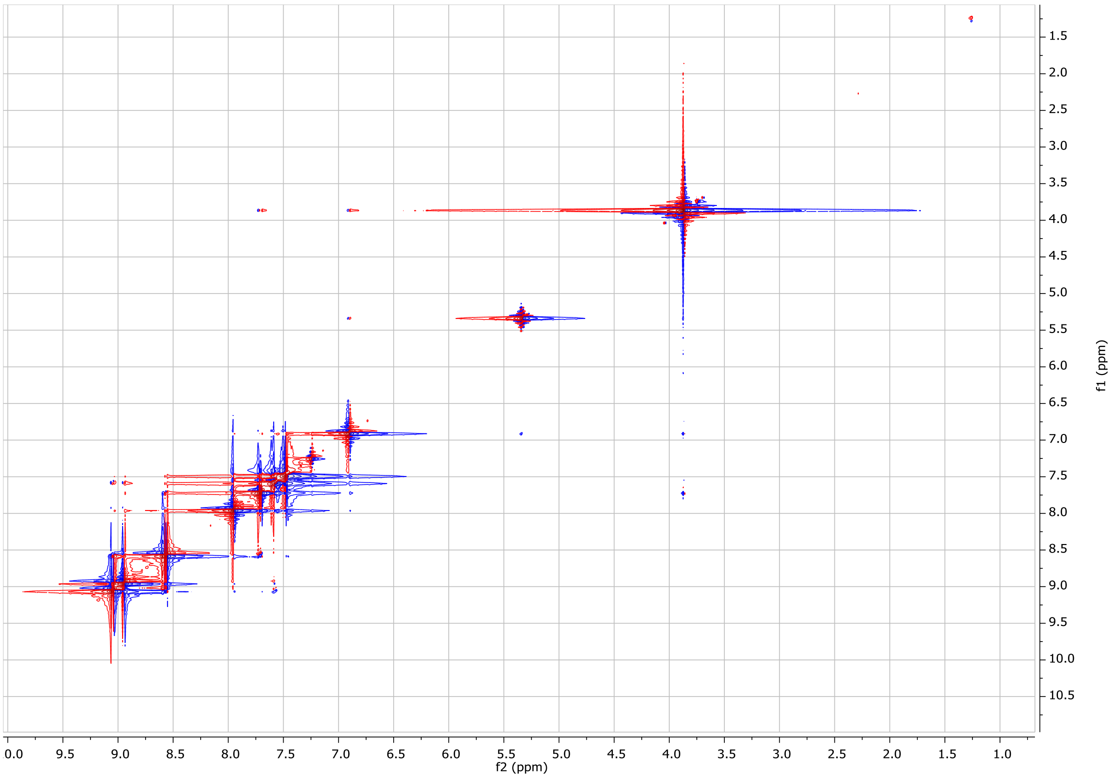

Figure S17. NOESY-Spectrum of **5a**

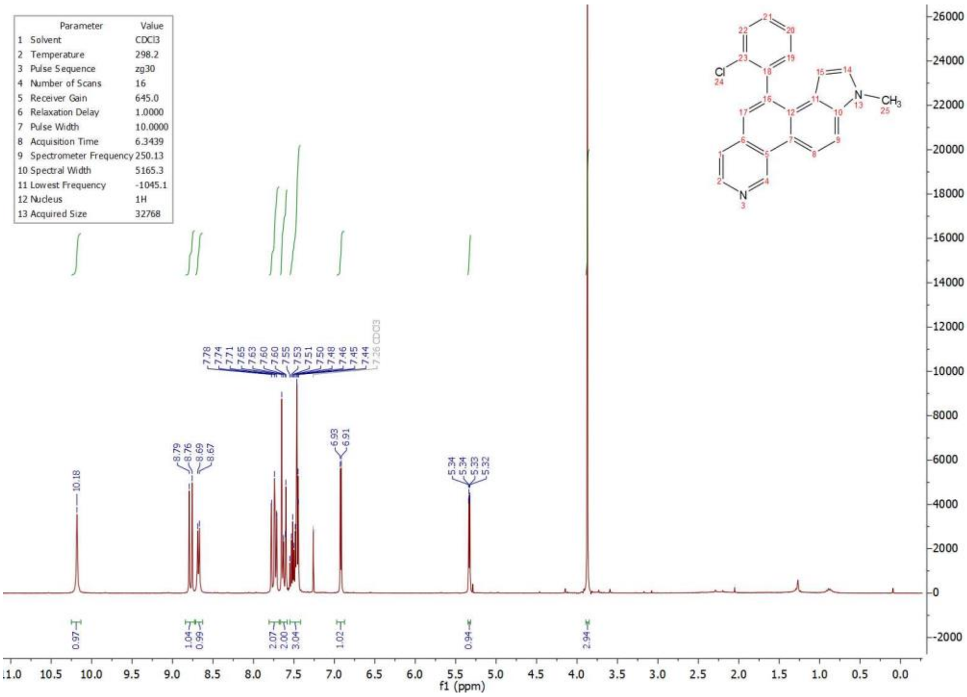

Figure S18. ^{1}H-NMR-spectrum of **5b**

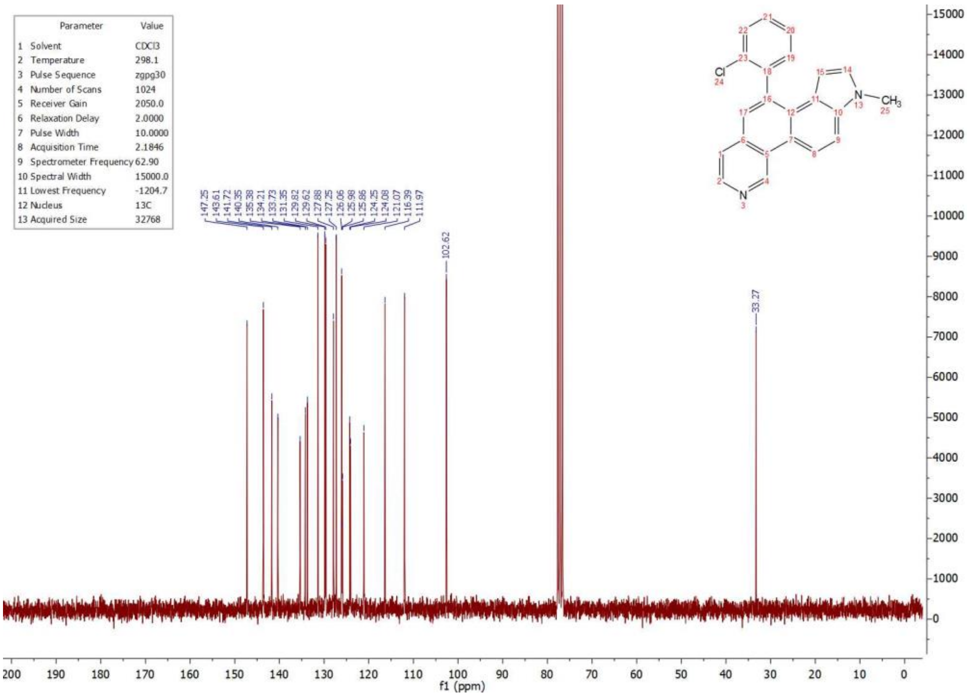

Figure S19. ^{13}C-NMR-spectrum of **5b**

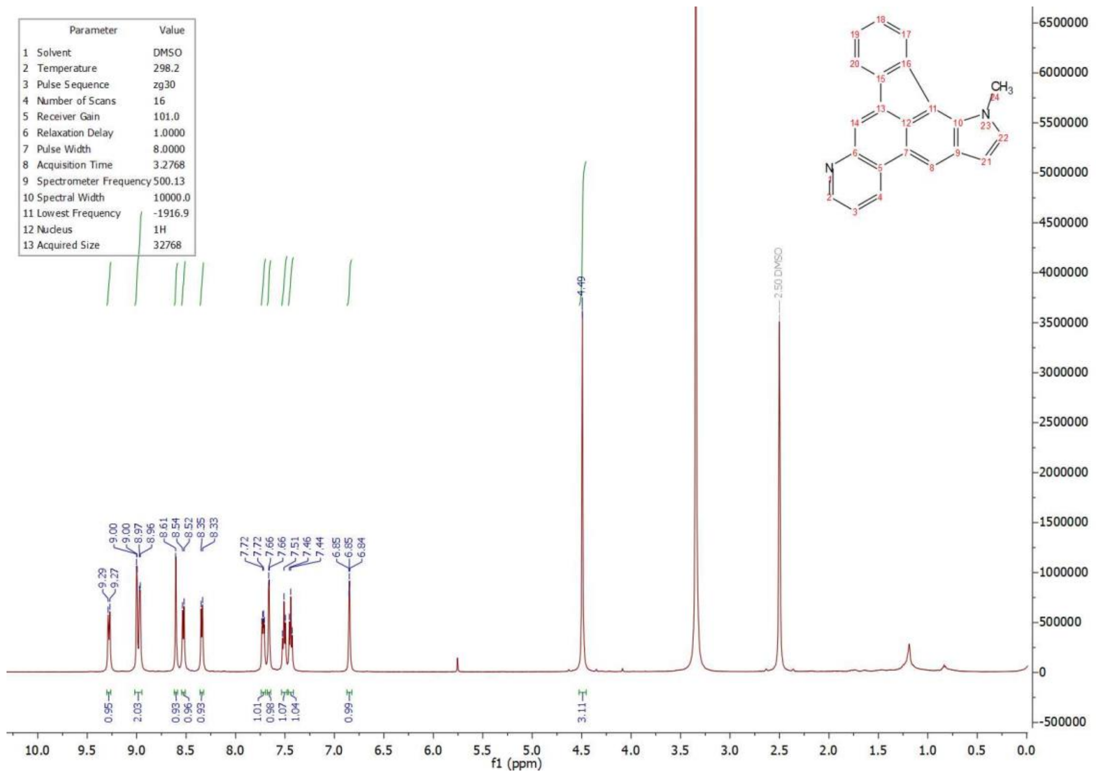

Figure S20. ^{1}H-NMR-spectrum of **6a**

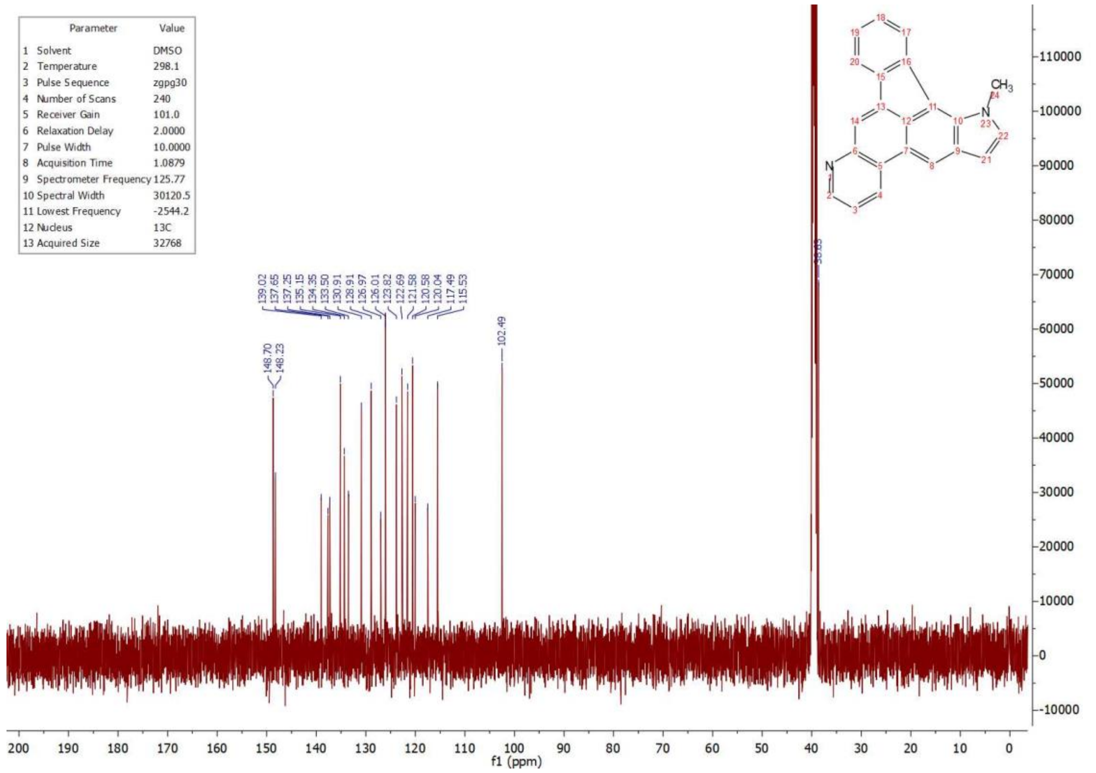

Figure S21. ^{13}C-NMR-spectrum of **6a**

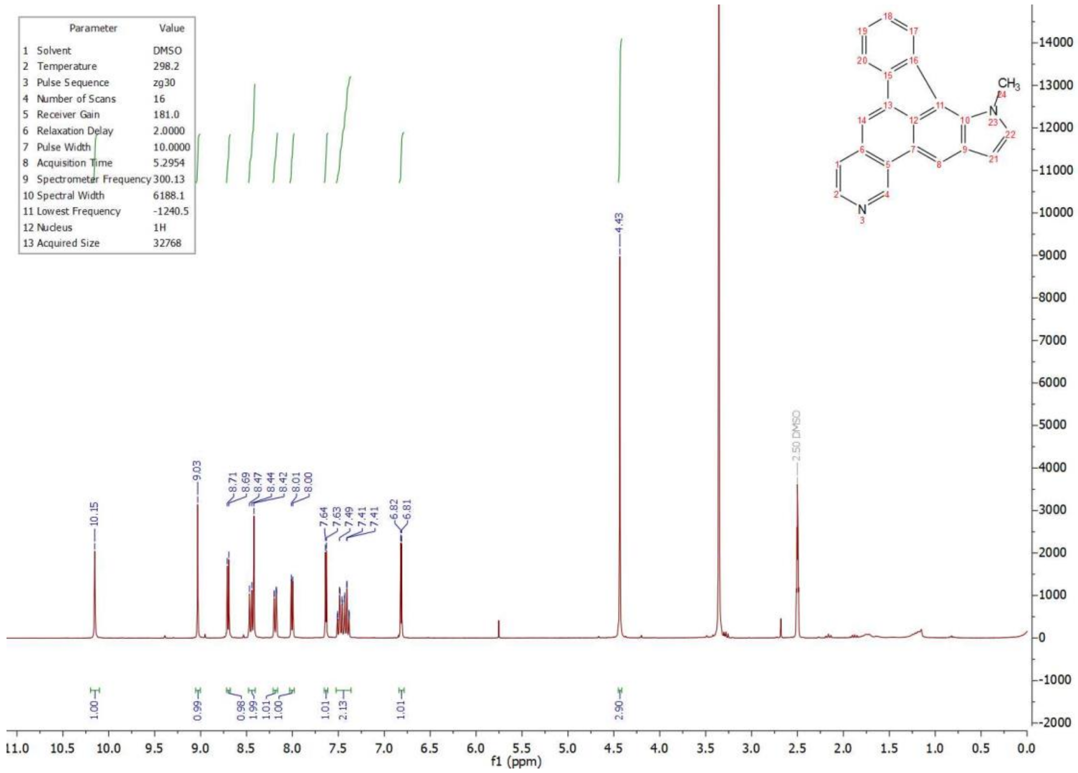

Figure S22. ^{1}H-NMR-spectrum of **6b**

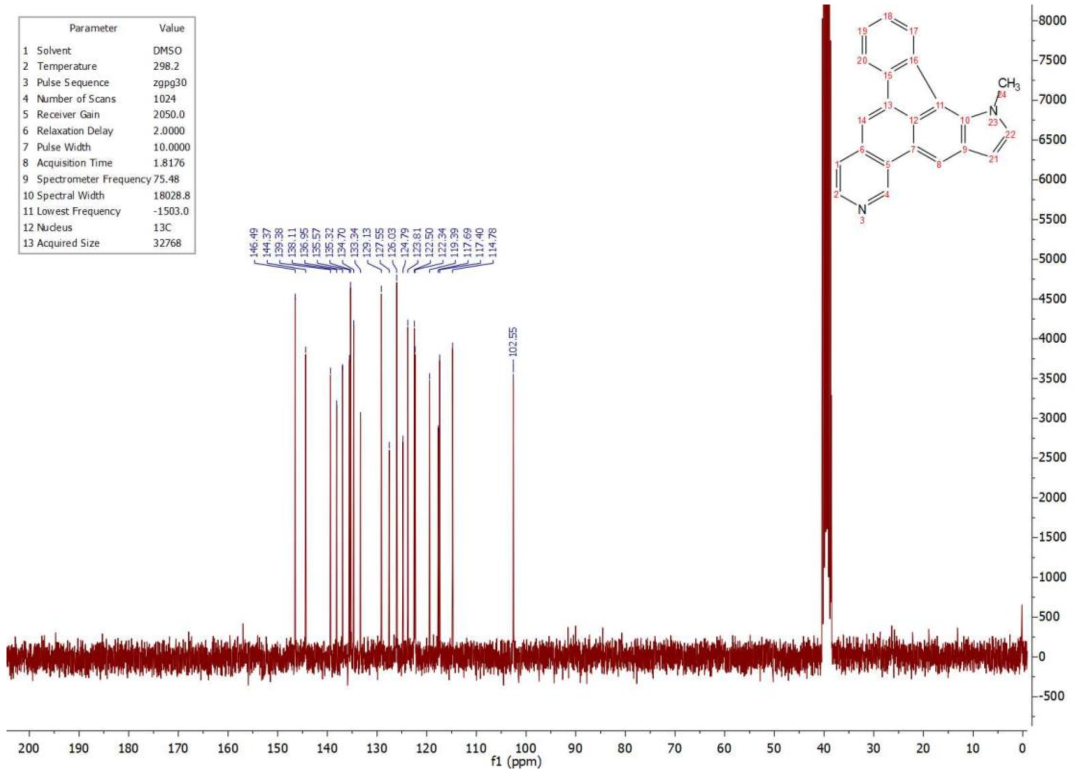

Figure S23. ^{13}C-NMR-spectrum of **6b**

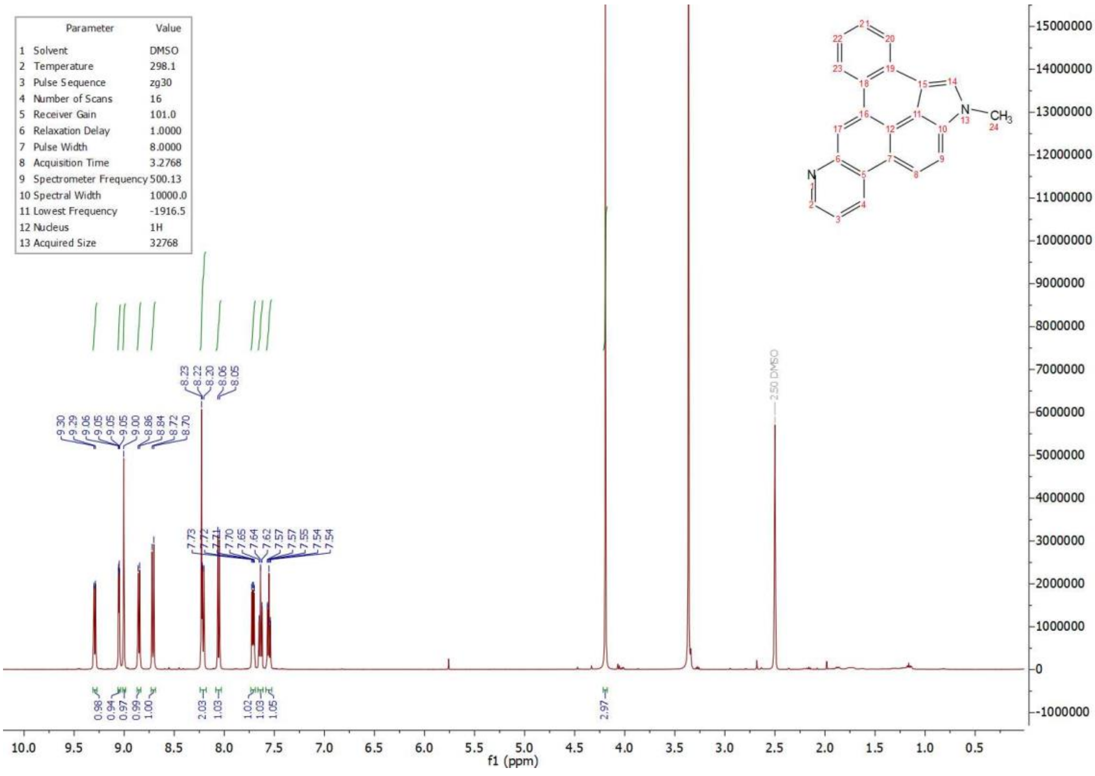

Figure S24. ^{1}H-NMR-spectrum of **7a**

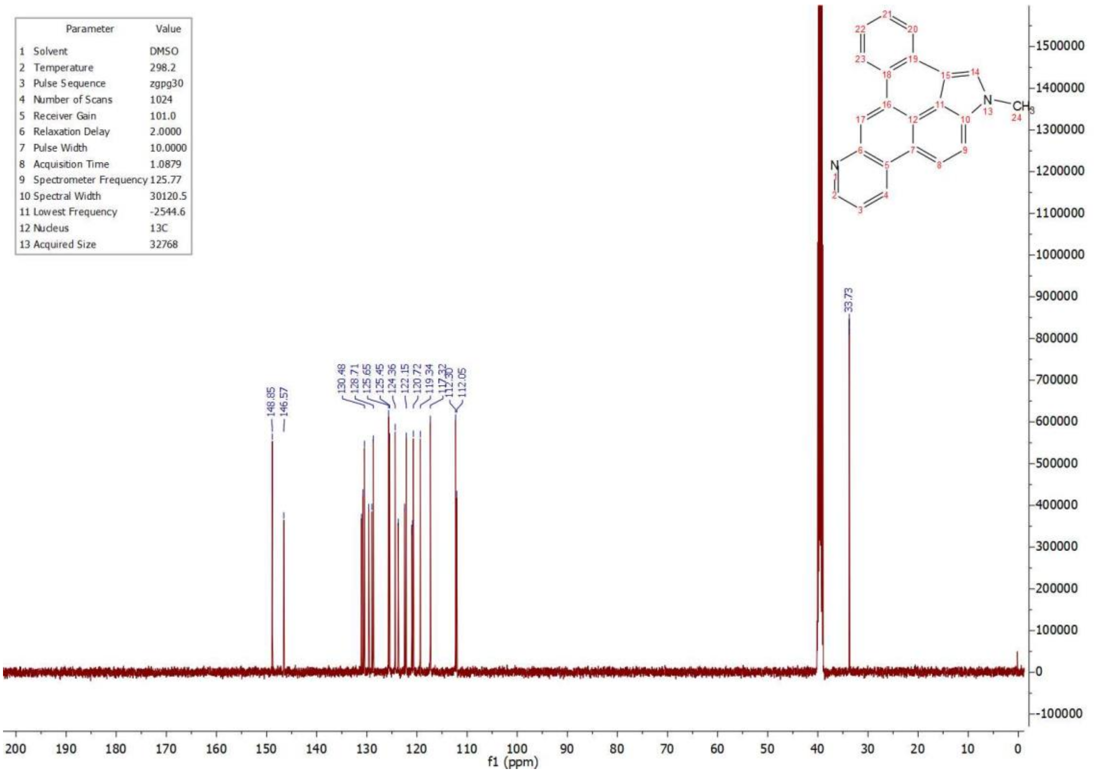

Figure S25. ^{13}C-NMR-spectrum of **7a**

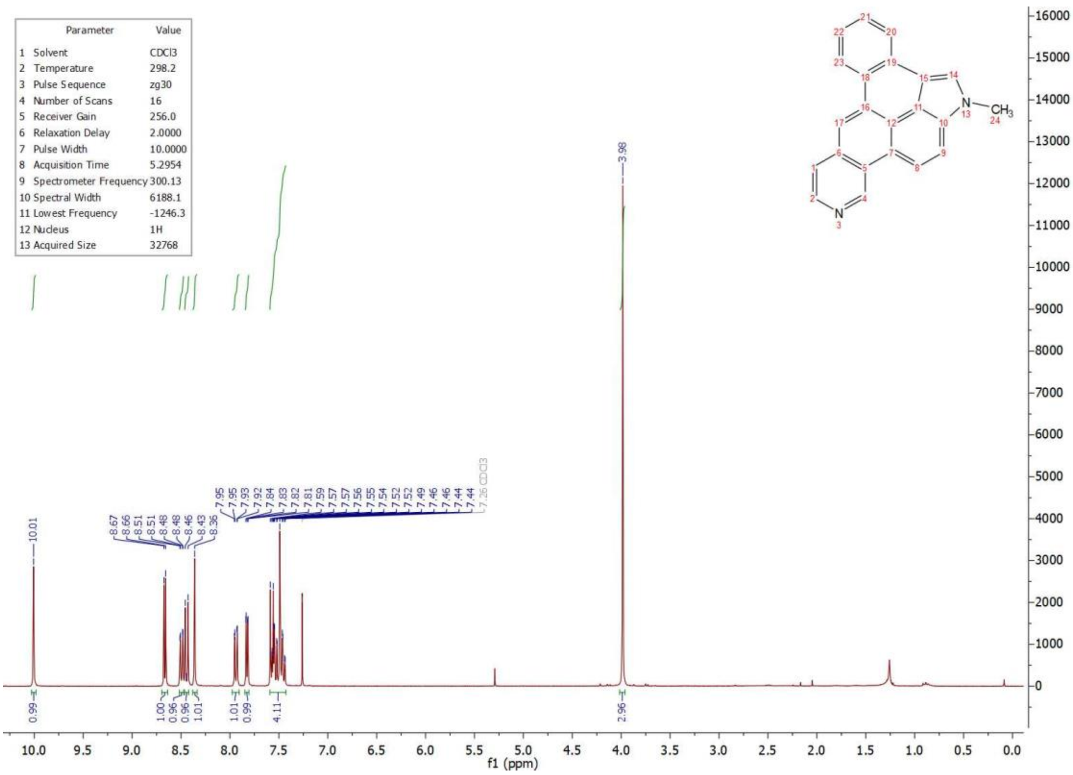

Figure S26. ^{1}H-NMR-spectrum of **7b**

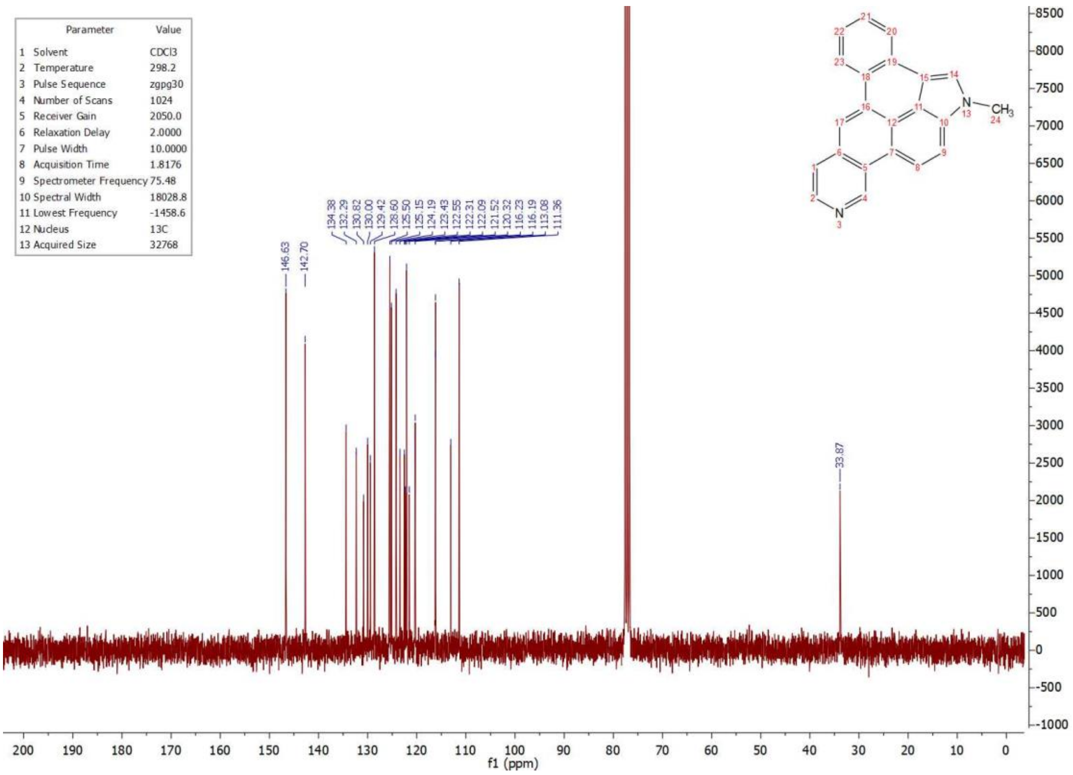

Figure S27. ^{13}C-NMR-spectrum of **7b**
